# Exact Expressions of Spin-Spin Correlation Functions of the Two-Dimensional Rectangular Ising Model on a Finite Lattice

**DOI:** 10.3390/e20040277

**Published:** 2018-04-12

**Authors:** Tao Mei

**Affiliations:** Department of Journal, Central China Normal University, Wuhan 430079, China; meitao@mail.ccnu.edu.cn

**Keywords:** two-dimensional Ising model, spin-spin correlation functions, exact solution, short-range order, long-range order

## Abstract

We employ the spinor analysis method to evaluate exact expressions of spin-spin correlation functions of the two-dimensional rectangular Ising model on a finite lattice, special process enables us to actually carry out the calculation process. We first present some exact expressions of correlation functions of the model with periodic-periodic boundary conditions on a finite lattice. The corresponding forms in the thermodynamic limit are presented, which show the short-range order. Then, we present the exact expression of the correlation function of the two farthest pair of spins in a column of the model with periodic-free boundary conditions on a finite lattice. Again, the corresponding form in the thermodynamic limit is discussed, from which the long-range order clearly emerges as the temperature decreases.

## 1. Introduction

Since the exact solution of the partition function in the absence of a magnetic field of the two-dimensional rectangular Ising model with periodic-periodic boundary conditions is obtained in the thermodynamic limit [1] and in finite-size systems [2], many authors have contributed to the knowledge of various aspects of this model, such as different boundary conditions, the arrangement modes of the spin lattice, surfaces, or mathematical methods, etc. [3,4,5,6,7].

Besides the partition function of the model, the calculations of spin-spin correlation functions are an important subject in the research of the two-dimensional Ising model. Some expressions of correlation functions in the thermodynamic limit have been obtained [3,4,5,8,9], and the case in a finite lattice has been studied [10,11,12].

The determination of exact expressions of the partition function and spin-spin correlation functions of the model on a finite lattice is not only a theoretical subject; the results obtained can also be used in the research of finite-size scaling, finite-size corrections, and boundary effects [7,13,14,15,16].

In this paper, we present some exact expressions of spin-spin correlation functions of the two-dimensional rectangular Ising model on a finite lattice by employing the spinor analysis method [2].

In Section 2, for the model with L rows and N columns and periodic-periodic boundary conditions (Onsager’s lattice), we calculate some exact expressions of the correlation function $\langle \sigma_{1\;,\;1}\sigma_{1\;,\;1+Q}\rangle$ and compare the corresponding forms in the thermodynamic limit obtained here with known results presented in Reference [9]. The investigation in Section 2 shows the main steps, key points, and problems of the approach used in this paper. Since the whole process are complex, here we outline the approach.

(1) Although any spin-spin correlation functions $\langle \sigma_{l\;,\;n}\sigma_{l^{\prime }\;,\;n^{\prime }}\rangle$ can be expressed by matrices, and the matrices belong to spin representatives [8], here we only consider $\langle \sigma_{l\;,\;1}\sigma_{l\;,\;1+Q}\rangle$, i.e., the correlation functions of pairwise spins in one column, of which exact expressions can be obtained by the spinor analysis method.

(2) When we employ the spinor analysis method to evaluate $\langle \sigma_{l\;,\;1}\sigma_{l\;,\;1+Q}\rangle$, it is very difficult to find the exact eigenvalues of the corresponding rotation matrix; however, the operators of the first derivative and limit in the expressions of $\langle \sigma_{l\;,\;1}\sigma_{l\;,\;1+Q}\rangle$ (see (8)) allow us to obtain exact expressions of $\langle \sigma_{1\;,\;1}\sigma_{1\;,\;1+Q}\rangle$ by only finding approximate eigenvalues of the rotation matrix.

(3) We employ Rayleigh-Schrodinger Perturbation Theory (RSPT) in quantum mechanics and change “finding eigenvalue up to Q-th order” to “finding eigenvalue through Q times first-order approximation” (see the discussions in Section 2.5) to find approximate eigenvalues of the rotation matrix. On the one hand, this approximate method enables us to actually carry out the calculation process. On the other hand, since RSPT is irregular, the approximate method is in fact incapable when *Q* is very larger. Hence, by this approach we can only obtain exact expressions of correlation functions when *Q* is a small number, for example, that of $\langle \sigma_{1\;,\;1}\sigma_{1\;,\;2}\rangle \;,$ $\langle \sigma_{1\;,\;1}\sigma_{1\;,\;3}\rangle \;,$ $\langle \sigma_{1\;,\;1}\sigma_{1\;,\;4}\rangle \;,\;\cdots$, etc., which belong to the short-range order.

What is more interesting is the long-range order, as it is closely related to properties of the phase transformation of the system. To obtain the correlation function that can reveal the long-range order of the model on a finite lattice, we turn to the model with L rows and N columns and periodic boundary condition in the horizontal direction and free boundary condition in the vertical direction. For this model, because of the free boundary condition in a column, and $\sigma_{l\;,\;N}$ is the farthest spin of $\sigma_{l\;,\;1}$ in the column, we forecast that the correlation functions $\langle \sigma_{l\;,\;1}\sigma_{l\;,\;N}\rangle$, $\langle \sigma_{l\;,\;1}\sigma_{l\;,\;N-1}\rangle$, $\langle \sigma_{l\;,\;1}\sigma_{l\;,\;N-2}\rangle \;,\;\cdots$ will display the long-range order.

For the model with periodic-free boundary conditions, if we write $\langle \sigma_{l\;,\;1}\sigma_{l\;,\;1+Q}\rangle$ in matrix forms and use the method presented in Section 2, then we still can only obtain exact expressions of $\langle \sigma_{1\;,\;1}\sigma_{1\;,\;2}\rangle \;,$ $\langle \sigma_{1\;,\;1}\sigma_{1\;,\;3}\rangle \;,\;\cdots$ but cannot obtain that of $\langle \sigma_{l\;,\;1}\sigma_{l\;,\;N}\rangle$, $\langle \sigma_{l\;,\;1}\sigma_{l\;,\;N-1}\rangle \;,\;\cdots$. On the other hand, if we write $\langle \sigma_{1\;,\;1}\sigma_{1\;,\;N-n}\rangle$ in matrix forms directly (see Formulas (52) and (53) in this paper), then the method presented in Section 2 is feasible to deal with the matrix forms of $\langle \sigma_{1\;,\;1}\sigma_{1\;,\;N-n}\rangle$, and we therefore can obtain some exact expressions of $\langle \sigma_{l\;,\;1}\sigma_{l\;,\;N}\rangle$, $\langle \sigma_{l\;,\;1}\sigma_{l\;,\;N-1}\rangle$, $\langle \sigma_{l\;,\;1}\sigma_{l\;,\;N-2}\rangle \;,\;\cdots$. 

However, to save space, in this paper we no longer discuss $\langle \sigma_{l\;,\;1}\sigma_{l\;,\;N-1}\rangle$, $\langle \sigma_{l\;,\;1}\sigma_{l\;,\;N-2}\rangle \;,\;\cdots$ but only evaluate $\langle \sigma_{l\;,\;1}\sigma_{l\;,\;N}\rangle$, for which all of the matrix forms, the corresponding rotation matrices, and the eigenvalue equations have been given by Reference [12]. Therefore, we only need to derive the exact expression of $\langle \sigma_{l\;,\;1}\sigma_{l\;,\;N}\rangle$ by employing the method presented in Section 2. (The reason why the determination of the exact expression of $\langle \sigma_{l\;,\;1}\sigma_{l\;,\;N}\rangle$ fails in Reference [12] is explained in Section 3.3).

After obtaining the exact expression of $\langle \sigma_{l\;,\;1}\sigma_{l\;,\;N}\rangle$, we discuss the properties of the expression of $\langle \sigma_{l\;,\;1}\sigma_{l\;,\;N}\rangle$ in the thermodynamic limit, from which the long-range order emerges as the temperature decreases, as shown clearly.

## 2. Short-Range Order in Onsager’s Lattice

### 2.1. The Definition of the Spin-Spin Correlation Functions and Their Basic Properties

According to the definition of the spin-spin correlation functions, $\langle \sigma_{l,\;n}\sigma_{l+P,\;n+Q}\rangle$ of pairwise spins $\sigma_{l\;,\;n}$ and $\sigma_{l+P\;,\;n+Q}$ of Onsager’s lattice read:

(1) $$\langle \sigma_{l,\;n}\sigma_{l+P,\;n+Q}\rangle =\frac{1}{Z}\sum_{\left\{\sigma_{h\;,\;v}=\pm 1\right\}}\sigma_{l,\;n}\sigma_{l+P,\;n+Q}\exp\left(\frac{J^{\prime }}{kT}\sum_{l^{\prime }=1}^{L}\sum_{n^{\prime }=1}^{N}\sigma_{l^{\prime }\;,\;n^{\prime }}\sigma_{l^{\prime }+1\;,\;n^{\prime }}\right)\exp\left(\frac{J}{kT}\sum_{l^{\prime }=1}^{L}\sum_{n^{\prime }=1}^{N}\sigma_{l^{\prime }\;,\;n^{\prime }}\sigma_{l^{\prime }\;,\;n^{\prime }+1}\right)\;,$$

where $\sigma_{L+1\;,\;n}=\sigma_{1\;,\;n}$, $\sigma_{l,\;N+1}=\sigma_{l\;,\;1}$; $J^{\prime }(>0)$, and J(>0) are the interaction constants for the horizontal and vertical directions, respectively;

(2) $$Z=\sum_{\left\{\sigma_{h\;,\;v}=\pm 1\right\}}\exp\left(\frac{J^{\prime }}{kT}\sum_{l=1}^{L}\sum_{n=1}^{N}\sigma_{l\;,\;n}\sigma_{l+1\;,\;n}\right)\exp\left(\frac{J}{kT}\sum_{l=1}^{L}\sum_{n=1}^{N}\sigma_{l\;,\;n}\sigma_{l\;,\;n+1}\right)$$

is the partition function in absence of a magnetic field of the model.

According to the periodic-periodic boundary conditions of Onsager’s lattice, it is easy to prove $\langle \sigma_{l,n}\sigma_{l+P,n+Q}\rangle =\langle \sigma_{1,1}\sigma_{1+P,1+Q}\rangle$; in this paper, we calculate $\langle \sigma_{l,n}\sigma_{l,n+Q}\rangle =\langle \sigma_{1,1}\sigma_{1,1+Q}\rangle$, i.e., we only calculate correlation functions of pairwise spins in one column.

Further, because of periodic-periodic boundary conditions, both $\sigma_{1\;,\;2}$ and $\sigma_{1\;,\;N}$ are the closest spins of $\sigma_{1\;,\;1}$, and we therefore have $\langle \sigma_{1,\;1}\sigma_{1,\;2}\rangle =\langle \sigma_{1,\;1}\sigma_{1,\;N}\rangle$. Generally speaking, it is easy to prove $\langle \sigma_{1,\;1}\sigma_{1,\;1+Q}\rangle =\langle \sigma_{1,\;1}\sigma_{1,\;1+N-Q}\rangle$ in terms of periodic-periodic boundary conditions. Hence, $\sigma_{1\;,\;1+[N/2]}$ is the farthest spin of $\sigma_{1\;,\;1}$, where [x] denotes the greatest integer not exceeding x. Thus, we only need to calculate $\langle \sigma_{1,\;1}\sigma_{1,\;1+Q}\rangle$ for $Q=1,\;2,\;\cdots ,\;\left[\frac{N}{2}\right]$.

### 2.2. Some Results Concerning the Partition Function Z

From (1), we see that to obtain $\langle \sigma_{1,\;1}\sigma_{1,\;1+Q}\rangle$, we need knowledge about the partition function Z given by (2), from which we summarize some results presented in Reference [2] as follows.

(3) $$Z={\left(2\sinh\frac{2J^{\prime }}{kT}\right)}^{\frac{LN}{2}}\tilde{Z},\;\tilde{Z}=\mathrm{Tr}\left(\frac{1+\Gamma_{2N+1}}{2}U^{\left(↑\right)}+\frac{1-\Gamma_{2N+1}}{2}U^{\left(↓\right)}\right).$$

$\tilde{Z}$ can thus be obtained by finding the trace of a matrix, where $\Gamma_{2N+1}$ is the matrix **U** defined by (15.68) in Reference [17], and the matrices $\frac{1+\Gamma_{2N+1}}{2}U^{\left(↑\right)}$ and $\frac{1-\Gamma_{2N+1}}{2}U^{\left(↓\right)}$ can be diagonalized at the same time.

On the other hand, both matrices $U^{\left(↑\right)}$ and $U^{\left(↓\right)}$ are spin representatives. By $\omega^{\left(↑\right)}$ and $\omega^{\left(↓\right)}$ we denote the corresponding rotation matrices of $U^{\left(↑\right)}$ and $U^{\left(↓\right)}$, respectively; both $\omega^{\left(↑\right)}$ and $\omega^{\left(↓\right)}$ can be diagonalized:

(4) $$\begin{array}{l} {\left(\zeta^{\left(↑↓\right)}\right)}^{+}\omega^{\left(↑↓\right)}\zeta^{\left(↑↓\right)}=\lambda^{\left(↑↓\right)};\\ \lambda^{\left(↑↓\right)}=\mathrm{diag}\left[\begin{array}{cccc} \lambda_{1}^{\left(↑↓\right)} & \lambda_{2}^{\left(↑↓\right)} & \cdots & \lambda_{N}^{\left(↑↓\right)} \end{array}\right]\;,\;\lambda_{n}^{\left(↑↓\right)}=\mathrm{diag}\left[\begin{array}{cc} e^{\gamma_{n}^{\left(↑↓\right)}} & e^{-\gamma_{n}^{\left(↑↓\right)}} \end{array}\right](n=1,\;2,\;\cdots ,\;N)\;, \end{array}$$

where $A^{+}$ indicates a complex conjugate to a matrix A,

(5) $$\begin{array}{l} \cosh\gamma_{n}=\cosh2K^{\prime }\cosh2K-\cos\varphi_{n}\sinh2K^{\prime }\sinh2K\;,\;e^{-2K^{\prime }}=\tanh\frac{J^{\prime }}{kT}\;,\;K=\frac{J}{kT};\\ \varphi_{n}^{\left(↑\right)}=\frac{(2n-1)\pi }{N},\;\varphi_{n}^{\left(↓\right)}=\frac{2n\pi }{N}\;(n=1,\;2,\;\cdots ,\;N=2M)\;. \end{array}$$

In the above formulas, $\gamma_{n}$ and $\varphi_{n}$ are as the abbreviations for $\gamma_{n}^{\left(↑↓\right)}$ and $\varphi_{n}^{\left(↑↓\right)}$, respectively. To save space, we use these abbreviation as far as possible in this paper.

Since properties of the partition function Z vary between even and odd numbers N, we must calculate $\langle \sigma_{1,\;1}\sigma_{1,\;1+Q}\rangle$ separately in terms of whether N is an even or odd number. In this paper we only consider the case that N=2M as an even number (the case of N=2M+1 can be dealt with by the same approach). From Reference [2], we have:

(6) $$\begin{array}{l} \tilde{Z}=\frac{1}{2}({\left(\prod_{m=1}^{M}2\cosh\frac{L\gamma_{m}^{\left(↑\right)}}{2}\right)}^{2}+{\left(\prod_{m=1}^{M}2\sinh\frac{L\gamma_{m}^{\left(↑\right)}}{2}\right)}^{2}\\ \;+\left(2\cosh\frac{L\gamma_{2M}^{\left(↓\right)}}{2}\right)\;\left(2\cosh\frac{L\gamma_{M}^{\left(↓\right)}}{2}\right)\;{\left(\prod_{m=1}^{M-1}2\cosh\frac{L\gamma_{m}^{\left(↓\right)}}{2}\right)}^{2}\\ \;+\left(2\sinh\frac{L\gamma_{2M}^{\left(↓\right)}}{2}\right)\;\left(2\sinh\frac{L\gamma_{M}^{\left(↓\right)}}{2}\right)\;{\left(\prod_{m=1}^{M-1}2\sinh\frac{L\gamma_{m}^{\left(↓\right)}}{2}\right)}^{2}). \end{array}$$

When N=2M, $\gamma_{n}$ and $\varphi_{n}$ have properties:

(7) $$\begin{array}{l} \gamma_{m}=\gamma_{m^{\prime }},\;\varphi_{m}=2\pi -\varphi_{m^{\prime }},\;m^{\prime }=\left\{ \begin{array}{ll} 2M-(m-1)\;, & \mathrm{for}\;(↑)\;;\\ 2M-m\;, & \mathrm{for}\;(↓)\;, \end{array}\right.m=\left\{ \begin{array}{ll} 1,\;2,\;\cdots ,\;M\;, & \mathrm{for}\;(↑)\;;\\ 1,\;2,\;\cdots ,\;M-1\;,\; & \mathrm{for}\;(↓)\;, \end{array}\right.\\ \gamma_{M}^{\left(↓\right)}=2(K+K^{\prime })\;,\;\gamma_{2M}^{\left(↓\right)}=2(K-K^{\prime })\;,\;\varphi_{M}^{\left(↓\right)}=\pi \;,\;\varphi_{2M}^{\left(↓\right)}=2\pi \;,\\ 0<\gamma_{1}^{\left(↑\right)}<\gamma_{2}^{\left(↑\right)}<\cdots <\gamma_{M}^{\left(↑\right)}\;,\;0<\left|\gamma_{2M}^{\left(↓\right)}\right|<\gamma_{1}^{\left(↓\right)}<\gamma_{2}^{\left(↓\right)}<\cdots <\gamma_{M-1}^{\left(↓\right)}<\gamma_{M}^{\left(↓\right)}\;. \end{array}$$

### 2.3. Writing $\langle \sigma_{1,\;1}\sigma_{1,\;1+Q}\rangle$ in Matrix Form

Since $\sigma_{h\;,\;v}^{2}=1$, we have $\sigma_{1\;,\;1}\sigma_{1\;,\;1+Q}=\sigma_{1\;,\;1}\sigma_{1\;,\;2}\cdot \sigma_{1\;,\;2}\sigma_{1\;,\;3}\cdot \cdots \cdot \sigma_{1\;,\;Q-1}\sigma_{1\;,\;Q}\cdot \sigma_{1\;,\;Q}\sigma_{1\;,\;1+Q}$, and, further, considering $\sigma_{1\;,\;q}\sigma_{1\;,\;1+q}=\underset{\phi_{q}\to 0}{\lim}\frac{\partial e^{\phi_{q}\sigma_{1\;,\;q}\sigma_{1\;,\;1+q}}}{\partial \phi_{q}}$, according to (1), $\langle \sigma_{1,\;1}\sigma_{1,\;1+Q}\rangle$ can be written in the form:

(8) $$\begin{array}{l} \langle \sigma_{1\;,\;1}\sigma_{1\;,\;1+Q}\rangle =\frac{1}{Z}\left(\prod_{q=1}^{Q}\underset{\phi_{q}\to 0}{\lim}\frac{\partial }{\partial \phi_{q}}\right)Y_{Q}\;,\\ Y_{Q}=\sum_{\left\{\sigma_{h\;,\;v}=\pm 1\right\}}\exp\left(\sum_{q=1}^{Q}\phi_{q}\sigma_{1\;,\;q}\sigma_{1\;,\;1+q}\right)\exp\left(\frac{J^{\prime }}{kT}\sum_{l=1}^{L}\sum_{n=1}^{N}\sigma_{l\;,\;n}\sigma_{l+1\;,\;n}\right)\exp\left(\frac{J}{kT}\sum_{l=1}^{L}\sum_{n=1}^{N}\sigma_{l\;,\;n}\sigma_{l\;,\;n+1}\right)\;, \end{array}$$

Also, by a standard method [1,17] we can further write $Y_{Q}$ in the form:

(9) $$Y_{Q}={\left(2\sinh\frac{2J^{\prime }}{kT}\right)}^{\frac{LN}{2}}{\tilde{Y}}_{Q},\;{\tilde{Y}}_{Q}=\mathrm{Tr}\left(\frac{1+\Gamma_{2N+1}}{2}V^{\left(↑\right)}+\frac{1-\Gamma_{2N+1}}{2}V^{\left(↓\right)}\right).$$

${\tilde{Y}}_{Q}$ can thus be obtained by finding the trace of a matrix. Here we are not going to write out the explicit expressions of $V^{\left(↑\right)}$ and $V^{\left(↓\right)}$, but only point out that:

- The matrices $\frac{1+\Gamma_{2N+1}}{2}V^{\left(↑\right)}$ and $\frac{1-\Gamma_{2N+1}}{2}V^{\left(↓\right)}$ can be diagonalized at the same time.
- Both $V^{\left(↑\right)}$ and $V^{\left(↓\right)}$ are spin representatives, whose corresponding rotation matrices are $\zeta^{\left(↑\right)}H^{\left(↑\right)}{\left(\zeta^{\left(↑\right)}\right)}^{+}$ and $\zeta^{\left(↓\right)}H^{\left(↓\right)}{\left(\zeta^{\left(↓\right)}\right)}^{+}$, respectively, where ζ are introduced by (4),

(10) $$H=H^{\left(0\right)}+\sum_{q=1}^{Q}\sinh2\phi_{q}H^{\left(q\right)}+2\sum_{q=1}^{Q}\sinh^{2}\phi_{q}{H^{\prime }}^{\left(q\right)},$$

the forms of the matrices $H^{\left(0\right)}$, $H^{\left(q\right)}$, and ${H^{\prime }}^{\left(q\right)}$ can be expressed in terms of 2×2 blocks $H_{lm}^{\left(0\right)}$, $H_{lm}^{\left(q\right)}$ and ${H^{\prime }}_{lm}^{\left(q\right)}$, 1≤l, m≤N(=2M), given by:

(11) $$H_{lm}^{\left(0\right)}=\left[\begin{array}{cc} e^{L\gamma_{l}} & 0\\ 0 & e^{-L\gamma_{l}} \end{array}\right]\delta_{lm}\;,$$

(12) $$H_{lm}^{\left(q\right)}=e^{-iq\frac{(l-m)\pi }{M}}H_{lm}\;,\;H_{lm}=\frac{1}{N}e^{-i\frac{\theta_{l}-\theta_{m}}{2}}\left[\begin{array}{cc} e^{\frac{L(\gamma_{l}+\gamma_{m})}{2}}\cos\frac{\theta_{l}+\theta_{m}}{2} & -{\mathrm{ie}}^{\frac{L(\gamma_{l}-\gamma_{m})}{2}}\sin\frac{\theta_{l}+\theta_{m}}{2}\\ {\mathrm{ie}}^{-\frac{L(\gamma_{l}-\gamma_{m})}{2}}\sin\frac{\theta_{l}+\theta_{m}}{2} & -e^{-\frac{L(\gamma_{l}+\gamma_{m})}{2}}\cos\frac{\theta_{l}+\theta_{m}}{2} \end{array}\right]\;,$$

(13) $${H^{\prime }}_{lm}^{\left(q\right)}=e^{-iq\frac{(l-m)\pi }{M}}{H^{\prime }}_{lm}\;,\;{H^{\prime }}_{lm}=\frac{1}{N}e^{-i\frac{\theta_{l}-\theta_{m}}{2}}\left[\begin{array}{cc} e^{\frac{L(\gamma_{l}+\gamma_{m})}{2}}\cos\frac{\theta_{l}-\theta_{m}}{2} & {\mathrm{ie}}^{\frac{L(\gamma_{l}-\gamma_{m})}{2}}\sin\frac{\theta_{l}-\theta_{m}}{2}\\ {\mathrm{ie}}^{-\frac{L(\gamma_{l}-\gamma_{m})}{2}}\sin\frac{\theta_{l}-\theta_{m}}{2} & e^{-\frac{L(\gamma_{l}+\gamma_{m})}{2}}\cos\frac{\theta_{l}-\theta_{m}}{2} \end{array}\right].$$

In (12) and (13), the quantities $\theta_{n}$ are defined by:

(14) $$\begin{array}{l} \sinh\gamma_{n}\cos\theta_{n}=\cosh2K^{\prime }\sinh2K-\cos\varphi_{n}\sinh2K^{\prime }\cosh2K\;,\\ \sinh\gamma_{n}\sin\theta_{n}=\sin\varphi_{n}\sinh2K^{\prime }\;(n=1,\;2,\;\cdots ,\;N=2M)\;. \end{array}$$

When N=2M, $\theta_{n}$ have properties:

(15) $$\theta_{m}=2\pi -\theta_{m^{\prime }},\;\theta_{M}^{\left(↓\right)}=\theta_{2M}^{\left(↓\right)}=0\;.$$

In (15), the values of $m^{\prime }$ and m are exactly the same as those in (7).

We can first evaluate the eigenvalues of the rotation matrices $\zeta H\zeta^{+}$, and then obtain ${\tilde{Y}}_{Q}$ in terms of the spinor analysis method. Finally, we obtain $\langle \sigma_{1,\;1}\sigma_{1,\;1+Q}\rangle$ according to (8) and (9).

From (10) to (13), we see that H is a Hermitian conjugate matrix. Hence, the eigenvalues of both matrices $\zeta H\zeta^{+}$ and H are the same, and we therefore can only evaluate the eigenvalues of the rotation matrix H.

### 2.4. Basic Properties of the Eigenvalues and Eigenvectors of the Matrix H

Any rotation matrix **A** has the following property: If τ is an eigenvalue of **A**, then $\tau^{-1}$ is also an eigenvalue of **A** [2]. Since $\zeta H\zeta^{+}$ is a rotation matrix and the eigenvalues of $\zeta H\zeta^{+}$ and H are the same, H should have the same property. In this sub-section we prove this conclusion.

The eigen equation of H reads:

(16) HΨ=τΨ.

By $\Psi ={\left[\begin{array}{ccccc} \psi_{1} & \cdots & \psi_{n} & \cdots & \psi_{N} \end{array}\right]}^{T},\psi_{n}=\left[\begin{array}{c} \psi_{n}^{\Delta }\\ \psi_{n}^{\nabla } \end{array}\right]$, where $A^{T}$ means the transpose of a matrix **A**, we denote the eigenvector of H, and, introducing $C_{q}^{\Delta }$, $C_{q}^{\nabla }$ (q=1, 2, ⋯, Q) in terms of:

$$\begin{array}{l} \left[\begin{array}{c} \psi_{n}^{\Delta }\\ \psi_{n}^{\nabla } \end{array}\right]=e^{-i\frac{\theta_{n}}{2}}\left[\begin{array}{cc} e^{-\frac{L\gamma_{n}}{2}} & 0\\ 0 & e^{\frac{L\gamma_{n}}{2}} \end{array}\right]\;\left[\begin{array}{cc} \cos\frac{\theta_{n}}{2} & \mathrm{isin}\frac{\theta_{n}}{2}\\ \mathrm{isin}\frac{\theta_{n}}{2} & \cos\frac{\theta_{n}}{2} \end{array}\right]\;\\ \;\times \left[\begin{array}{cc} \frac{\tau \;\cos h(L\gamma_{n})-1+\tau \;\cos\theta_{n}\sin h(L\gamma_{n})}{\tau^{2}-2\tau \cosh(L\gamma_{n})+1} & i\frac{\tau \;\sin\theta_{n}\sin h(L\gamma_{n})}{\tau^{2}-2\tau \cosh(L\gamma_{n})+1}\\ -i\frac{\tau \;\sin\theta_{n}\sin h(L\gamma_{n})}{\tau^{2}-2\tau \cosh(L\gamma_{n})+1} & \frac{\tau \;\cos h(L\gamma_{n})-1+\tau \;\cos\theta_{n}\sin h(L\gamma_{n})}{\tau^{2}-2\tau \cosh(L\gamma_{n})+1} \end{array}\right]\;\left[\begin{array}{c} \sum_{q=1}^{Q}e^{-iq\varphi_{n}}C_{q}^{\Delta }\\ \sum_{q=1}^{Q}e^{-iq\varphi_{n}}C_{q}^{\nabla } \end{array}\right]\;, \end{array}$$

we can prove that the eigen Equation (16) is equivalent to:

(17) $$\coth\phi_{q}\left[\begin{array}{c} C_{q}^{\Delta }\\ C_{q}^{\nabla } \end{array}\right]=\sum_{q^{\prime }=1}^{Q}\left[\begin{array}{cc} A_{+}(q-q^{\prime }) & -B(q-q^{\prime })\\ -B(q-q^{\prime }) & -A_{-}(q-q^{\prime }) \end{array}\right]\left[\begin{array}{c} C_{q^{\prime }}^{\Delta }\\ C_{q^{\prime }}^{\nabla } \end{array}\right]\;(q=1,\;2,\;\cdots ,\;Q)\;;$$

where

$$\begin{array}{l} A_{\pm }(k)=\frac{1}{N}\sum_{n=1}^{N}e^{ik\frac{2n\pi }{N}}\frac{\tau^{2}\pm 2\tau \;\cos\theta_{n}\sin h(L\gamma_{n})-1}{\tau^{2}-2\tau \cosh(L\gamma_{n})+1}\\ =\left\{ \begin{array}{ll} \frac{1}{M}\sum_{m=1}^{M}\cos\frac{km\pi }{M}\frac{\tau^{2}\pm 2\tau \;\cos\theta_{m}^{\left(↑\right)}\sin h(L\gamma_{m}^{\left(↑\right)})-1}{\left(e^{L\gamma_{m}^{\left(↑\right)}}\tau -1\right)\;\left(e^{-L\gamma_{m}^{\left(↑\right)}}\tau -1\right)}\;, & \mathrm{for}\;(↑)\;;\\ \frac{1}{M}\left(\frac{{\left(-1\right)}^{k}}{2}\frac{e^{\mp L\gamma_{M}^{\left(↓\right)}}\tau +1}{e^{\mp L\gamma_{M}^{\left(↓\right)}}\tau -1}+\frac{1}{2}\frac{e^{\mp L\gamma_{2M}^{\left(↓\right)}}\tau +1}{e^{\mp L\gamma_{2M}^{\left(↓\right)}}\tau -1}+\sum_{m=1}^{M-1}\cos\frac{km\pi }{M}\frac{\tau^{2}\pm 2\tau \;\cos\theta_{m}^{\left(↓\right)}\sin h(L\gamma_{m}^{\left(↓\right)})-1}{\left(e^{L\gamma_{m}^{\left(↓\right)}}\tau -1\right)\;\left(e^{-L\gamma_{m}^{\left(↓\right)}}\tau -1\right)}\right)\;, & \mathrm{for}\;(↓), \end{array}\right. \end{array}$$

$$B(k)=-i\frac{1}{N}\sum_{n=1}^{N}e^{ik\frac{2n\pi }{N}}\frac{2\tau \;\sin\theta_{n}\sin h(L\gamma_{n})}{\tau^{2}-2\tau \cosh(L\gamma_{n})+1}=\left\{ \begin{array}{ll} \frac{1}{M}\sum_{m=1}^{M}\sin\frac{km\pi }{M}\frac{2\tau \;\sin\theta_{m}^{\left(↑\right)}\sin h(L\gamma_{m}^{\left(↑\right)})}{\tau^{2}-2\tau \cosh(L\gamma_{m}^{\left(↑\right)})+1}, & \mathrm{for}\;(↑)\;;\\ \frac{1}{M}\sum_{m=1}^{M-1}\sin\frac{km\pi }{M}\frac{2\tau \;\sin\theta_{m}^{\left(↓\right)}\sin h(L\gamma_{m}^{\left(↓\right)})}{\tau^{2}-2\tau \cosh(L\gamma_{m}^{\left(↓\right)})+1}, & \mathrm{for}\;(↓), \end{array}\right.$$

where we have used (7) and (15). We see that all $A_{\pm }(k)$ and B(k) are real functions and satisfy:

$$A_{\pm }(-k)=A_{\pm }(k),\;B(-k)=-B(k).$$

According to the above expressions and the properties of $A_{\pm }(k)$ and B(k), we can conclude that if τ and $C_{q}^{\Delta }(\tau )\;,\;C_{q}^{\nabla }(\tau )\;(q=1,\;2,\;\cdots ,\;Q)$ satisfy (17), then $\tau^{\prime }=\tau^{-1}$ and

(18) $$C_{q}^{\Delta }(\tau^{\prime })=C_{0}(\tau )C_{q}^{\nabla }(\tau )\;,\;C_{q}^{\nabla }(\tau^{\prime })=C_{0}(\tau )C_{q}^{\Delta }(\tau )\;(q=1,\;2,\;\cdots ,\;Q)$$

also satisfy (17), where $C_{0}(\tau )$ is an arbitrary function. From this discussion we not only prove the conclusion “If τ is an eigenvalue of the matrix **H**, then $\tau^{-1}$ is also an eigenvalue”, but also obtain the relation (18) between $\left\{\;C_{q}^{\Delta }(\tau ),\;C_{q}^{\nabla }(\tau )\right\}$ and $\left\{\;C_{q}^{\Delta }(\tau^{-1})\;,\;C_{q}^{\nabla }(\tau^{-1})\right\}$. The conclusion and the relation (18) are useful to determine the forms of approximate eigenvalues and the expressions of the normalized eigenvectors of H, as well as to calculate the determinant of the matrix consisting of the eigenvectors in the actual calculation process.

### 2.5. Approximate Method for Solving the Eigen Equation (16)

It is very difficult to find the exact eigenvalues of H by solving the eigen Equation (16). On the other hand, the operator $\prod_{q=1}^{Q}\underset{\phi_{q}\to 0}{\lim}\frac{\partial }{\partial \phi_{q}}$ in (8) allows us to ignore all terms whose orders are higher than $\phi_{q}^{1}(=\phi_{q})$ (q=1, 2, ⋯, Q) in all eigenvalues of H. According to this key property, we can obtain the exact expressions of $\langle \sigma_{1,\;1}\sigma_{1,\;1+Q}\rangle$ by only finding approximate eigenvalues of H.

Concretely, as the first step, the term $2\sum_{q=1}^{Q}\sinh^{2}\phi_{q}{H^{\prime }}^{\left(q\right)}$ with the factors $\sinh^{2}\phi_{q}$ in (10) can be ignored, since $\sinh^{2}\phi_{q}\approx \phi_{q}^{2}$ have $\phi_{q}^{2}$ order. Then, from (11) we see that $H^{\left(0\right)}$ in (10) is a diagonal matrix, whose eigenvalues and eigenvectors are summarized in the following formulas:

(19) $$\begin{array}{l} H^{\left(0\right)}\Psi_{n,\;\pm }^{\left(0\right)}=e^{\pm L\gamma_{n}}\Psi_{n,\;\pm }^{\left(0\right)}\;(n=1,\;2,\;\cdots ,\;N=2M);\\ \Psi_{n,\;\pm }^{\left(0\right)}={\left[\begin{array}{ccccc} \psi_{n\;,\;1\;,\;\pm }^{\left(0\right)} & \cdots & \psi_{n\;,\;m\;,\;\pm }^{\left(0\right)} & \cdots & \psi_{n\;,\;N\;,\;\pm }^{\left(0\right)} \end{array}\right]}^{\;T},\;\psi_{n,\;m,\;\pm }^{\left(0\right)}=\left[\begin{array}{c} 0\\ 0 \end{array}\right]\;(m\neq n)\;,\\ \psi_{n,\;n,\;+}^{\left(0\right)}=\left\{ \begin{array}{l} \left[\begin{array}{c} 1\\ 0 \end{array}\right]\;,\;\mathrm{for}\;\mathrm{the}\;\mathrm{eigenvalue}\;e^{L\gamma_{n}};\\ \left[\begin{array}{c} 0\\ 0 \end{array}\right]\;,\;\mathrm{for}\;\mathrm{the}\;\mathrm{eigenvalue}\;e^{-L\gamma_{n}}, \end{array}\right.\psi_{n,\;n,\;-}^{\left(0\right)}=\left\{ \begin{array}{l} \left[\begin{array}{c} 0\\ 0 \end{array}\right]\;,\;\mathrm{for}\;\mathrm{the}\;\mathrm{eigenvalue}\;e^{L\gamma_{n}};\\ \left[\begin{array}{c} 0\\ 1 \end{array}\right]\;,\;\mathrm{for}\;\mathrm{the}\;\mathrm{eigenvalue}\;e^{-L\gamma_{n}}. \end{array}\right. \end{array}$$

which are as the zeroth order approximation of the eigen Equation (16).

The term $\sum_{q=1}^{Q}\sinh2\phi_{q}H^{\left(q\right)}$ with the factors $\sinh2\phi_{q}\approx 2\phi_{q}$ (q=1, 2, ⋯, Q) in (10) can be regarded as a perturbation term. Then, by using RSPT, we can obtain the approximate eigenvalues of H.

However, although what eigenvalues we need are only corrected to the $\phi_{q}^{1}(=\phi_{q})$ order (q=1, 2, ⋯, Q), we must calculate the perturbation terms up to the Q-th order, not only for the first-order approximation, because all of the terms with the factor $\prod_{q=1}^{Q}\sinh2\phi_{q}$ appear in the Q-th order eigenvalues and are needed, which only include the $\phi_{q}^{1}$ order for every $\phi_{q}$.

However, if we calculate the eigenvalues up to the Q-th order by using RSPT, then not only the actual calculation process is very complex, but there are also many unwanted terms with factors $\phi_{q}^{k}\;(k\geq 2)$, for example, the term with the factor $\sinh^{3}2\phi_{1}\prod_{q=4}^{Q}\sinh2\phi_{q}$, in the Q-th order eigenvalues.

To take out those terms with factors $\phi_{q}^{k}\;(k\geq 2)$, we change “finding the eigenvalue up to the Q-th order” to “finding the eigenvalue through Q times first-order approximation”. 

Concretely, since now $H=H^{\left(0\right)}+\sum_{q=1}^{Q}\sinh2\phi_{q}H^{\left(q\right)}$, we first consider the matrix $H^{\left(0\right)}+\sinh2\phi_{1}H^{\left(1\right)}$, in which the eigenvalues and eigenvectors ${\left\{\;\tau^{\left(0\right)},\;\Psi^{\left(0\right)}\right\}}^{}$ of $H^{\left(0\right)}$ are given by (19) and $\sinh2\phi_{1}H^{\left(1\right)}$ is as perturbation term. By only calculating first-order approximation we obtain all eigenvalues and eigenvectors ${\left\{\;\tau^{\left(1\right)},\;\Psi^{\left(1\right)}\right\}}^{}$ of $H^{\left(0\right)}+\sinh2\phi_{1}H^{\left(1\right)}$; therefore, all terms in ${\left\{\;\tau^{\left(1\right)},\;\Psi^{\left(1\right)}\right\}}^{}$ only correct to the $\phi_{1}^{1}$ order. 

Then, we consider the matrix $H^{\left(0\right)}+\sinh2\phi_{1}H^{\left(1\right)}+\sinh2\phi_{2}H^{\left(2\right)}$. Since now all eigenvalues and eigenvectors ${\left\{\;\tau^{\left(1\right)},\;\Psi^{\left(1\right)}\right\}}^{}$ of $H^{\left(0\right)}+\sinh2\phi_{1}H^{\left(1\right)}$ are known, we regard $\sinh2\phi_{2}H^{\left(2\right)}$ as a perturbation term, and, by only calculating the first-order approximation, obtain all eigenvalues and eigenvectors ${\left\{\;\tau^{\left(2\right)},\;\Psi^{\left(2\right)}\right\}}^{}$ of $H^{\left(0\right)}+\sinh2\phi_{1}H^{\left(1\right)}+\sinh2\phi_{2}H^{\left(2\right)}$, in which all terms are only of the $\phi_{1}^{1}$ and $\phi_{2}^{1}$ orders. In particular, all of the terms with the factor $\sinh2\phi_{1}\sinh2\phi_{2}\approx \phi_{1}\phi_{2}$ remain.

Then, we consider the matrix $H^{\left(0\right)}+\sinh2\phi_{1}H^{\left(1\right)}+\sinh2\phi_{2}H^{\left(2\right)}+\sinh2\phi_{3}H^{\left(3\right)}$. Since now all eigenvalues and eigenvectors ${\left\{\;\tau^{\left(2\right)},\;\Psi^{\left(2\right)}\right\}}^{}$ of $H^{\left(0\right)}+\sinh2\phi_{1}H^{\left(1\right)}+\sinh2\phi_{2}H^{\left(2\right)}$ are known, we regard $\sinh2\phi_{3}H^{\left(3\right)}$ as a perturbation term, and, by only calculating the first-order approximation, obtain all eigenvalues and eigenvectors ${\left\{\;\tau^{\left(3\right)},\;\Psi^{\left(3\right)}\right\}}^{}$ of $H^{\left(0\right)}+\sinh2\phi_{1}H^{\left(1\right)}+\sinh2\phi_{2}H^{\left(2\right)}+\sinh2\phi_{3}H^{\left(3\right)}$, in which all terms are only of the $\phi_{1}^{1}$, $\phi_{2}^{1}$ and $\phi_{3}^{1}$ orders. In particular, all of the terms with the factor $\sinh2\phi_{1}\sinh2\phi_{2}\sinh2\phi_{3}\approx \phi_{1}\phi_{2}\phi_{3}$ remain, and, many unwanted terms with factors $\sinh^{2}2\phi_{1}\sinh2\phi_{2}$, $\sinh2\phi_{2}\sinh^{2}2\phi_{3}$, etc., do not appear in the eigenvalues of $\tau^{\left(3\right)}$.

We follow this approach up to $\sinh2\phi_{Q}H^{\left(Q\right)}$ and every time we only calculate thr first-order approximation, which leads to the eigenvalues and eigenvectors ${\left\{\;\tau^{\left(Q\right)},\;\Psi^{\left(Q\right)}\right\}}^{}$ of all terms being only of the $\phi_{q}^{1}\left(=\phi_{q}\right)$ order. All of the terms with $\prod_{q=1}^{Q}\sinh2\phi_{q}$ remain, and at the same time those unwanted terms with $\phi_{q}^{k}\;(k\geq 2)$ do not appear.

On the one hand, the above approximate method allows us to actually carry out the calculation process to find the eigenvalues and eigenvectors of H. In particular, once we obtain ${\left\{\;\tau^{\left(1\right)},\;\Psi^{\left(1\right)}\right\}}^{}$, we can obtain ${\tilde{Y}}_{1}$ by the spinor analysis method, as well as obtain $\langle \sigma_{1,\;1}\sigma_{1,\;2}\rangle$ in terms of (8) and (9). Once we obtain ${\left\{\;\tau^{\left(2\right)},\;\Psi^{\left(2\right)}\right\}}^{}$, we can obtain ${\tilde{Y}}_{2}$ by the spinor analysis method, and, further, obtain $\langle \sigma_{1,\;1}\sigma_{1,\;3}\rangle$ in terms of (8) and (9),⋯. Generally speaking, once we obtain ${\left\{\;\tau^{\left(q\right)},\;\Psi^{\left(q\right)}\right\}}^{}$, we can obtain ${\tilde{Y}}_{q}$, and, further, obtain $\langle \sigma_{1,\;1}\sigma_{1,\;1+q}\rangle$.

On the other hand, since RSPT is irregular, when *Q* is very large, e.g., $Q\approx \left[\frac{N}{2}\right]$, the above approach no longer functions. Hence, by this approach we can only obtain the exact expressions of correlation functions when *Q* is a small number, for example, $\langle \sigma_{1,\;1}\sigma_{1,\;2}\rangle \;,$ $\langle \sigma_{1,\;1}\sigma_{1,\;3}\rangle \;,$ $\langle \sigma_{1,\;1}\sigma_{1,\;4}\rangle \;,\;\cdots$, etc., which belong to the short-range order, but we cannot obtain the exact expressions of correlation functions when *Q* is larger, for example, $\langle \sigma_{1,\;1}\sigma_{1,\;[N/2]+1}\rangle$, $\langle \sigma_{1,\;1}\sigma_{1,\;[N/2]}\rangle$, $\langle \sigma_{1,\;1}\sigma_{1,\;[N/2]-1}\rangle \;,\;\cdots$, etc., which belong to the long-range order.

### 2.6. Recurrence Formulas of the Eigenvalues and Eigenvectors $\left\{\tau^{\left(Q\right)},\;\Psi^{\left(Q\right)}\right\}$

According to the discussions in the above sub-section, we first regard $\sinh2\phi_{1}H^{\left(1\right)}$ as a perturbation term, and, by using RSPT, evaluate eigenvalues and eigenvectors ${\left\{\;\tau^{\left(1\right)},\;\Psi^{\left(1\right)}\right\}}^{}$ of the matrix $H^{\left(0\right)}+\sinh2\phi_{1}H^{\left(1\right)}$ up to the first-order approximation. However, according to (7), all eigenvalues $e^{\pm L\gamma_{n}^{\left(↑\right)}}$ are doubly-degenerate; and, except $e^{\pm L\gamma_{2M}^{\left(↓\right)}}$ and $e^{\pm L\gamma_{M}^{\left(↓\right)}}$, all the remaining eigenvalues $e^{\pm L\gamma_{n}^{\left(↓\right)}}$ are also doubly-degenerate. Hence, for doubly-degenerate eigenvalues of $H^{\left(0\right)}$, we must use the degenerate perturbation theory; the results obtained up to $\phi_{1}^{1}$ order are as follows.

(20) $$\alpha_{m\;,\;↑↓}^{\left(1\right)}=\frac{\sinh2\phi_{1}}{M}\cos^{2}\frac{\theta_{m}^{\left(↑↓\right)}}{2}\;,\;\beta_{m\;,\;↑↓}^{\left(1\right)}=\frac{\sinh2\phi_{1}}{M}\sin^{2}\frac{\theta_{m}^{\left(↑↓\right)}}{2}\;,\;\delta_{M}^{\left(1\right)}=\delta_{2M}^{\left(1\right)}=\frac{\sinh2\phi_{1}}{2M},$$

(21) $$\begin{array}{l} \Delta \Psi_{m,\;\pm ,\;↑↓,\;I}^{\left(0\right)}=\frac{\cos\frac{\theta_{m}^{\left(↑↓\right)}}{2}}{\sqrt{2}}\left(\sum_{\begin{array}{l} l=1\\ l\neq m\;,\;l\neq m^{\prime } \end{array}}^{2M}\frac{\cos\frac{\theta_{l}^{\left(↑↓\right)}}{2}}{\sin h\frac{L\left(\gamma_{l}^{\left(↑↓\right)}-\gamma_{m}^{\left(↑↓\right)}\right)}{2}}\Psi_{l,\;\pm }^{\left(0\right)}-\sum_{l=1}^{2M}\frac{i\sin\frac{\theta_{l}^{\left(↑↓\right)}}{2}}{\sinh\frac{L\left(\gamma_{l}^{\left(↑↓\right)}+\gamma_{m}^{\left(↑↓\right)}\right)}{2}}\Psi_{l,\;\mp }^{\left(0\right)}\right)\;,\\ \Delta \Psi_{m,\;\pm ,\;↑↓,\;\mathrm{II}}^{\left(0\right)}=\frac{i\sin\frac{\theta_{m}^{\left(↑↓\right)}}{2}}{\sqrt{2}}\left(\sum_{\begin{array}{l} l=1\\ l\neq m\;,\;l\neq m^{\prime } \end{array}}^{2M}\frac{i\sin\frac{\theta_{l}^{\left(↑↓\right)}}{2}}{\sin h\frac{L\left(\gamma_{l}^{\left(↑↓\right)}-\gamma_{m}^{\left(↑↓\right)}\right)}{2}}\Psi_{l,\;\pm }^{\left(0\right)}-\sum_{l=1}^{2M}\frac{\cos\frac{\theta_{l}^{\left(↑↓\right)}}{2}}{\sinh\frac{L\left(\gamma_{l}^{\left(↑↓\right)}+\gamma_{m}^{\left(↑↓\right)}\right)}{2}}\Psi_{l,\;\mp }^{\left(0\right)}\right)\;,\\ \Delta \Psi_{M,\;\pm ,\;↓}^{\left(0\right)}=\sum_{\begin{array}{l} \;l=1\\ l\neq M \end{array}}^{2M}\frac{\cos\frac{\theta_{l}^{\left(↓\right)}}{2}}{\sin h\frac{L\left(\gamma_{l}^{\left(↓\right)}-\gamma_{M}^{\left(↓\right)}\right)}{2}}\Psi_{l,\;\pm }^{\left(0\right)}-\sum_{l=1}^{2M}\frac{i\sin\frac{\theta_{l}^{\left(↓\right)}}{2}}{\sinh\frac{L\left(\gamma_{l}^{\left(↓\right)}+\gamma_{M}^{\left(↓\right)}\right)}{2}}\Psi_{l,\;\mp }^{\left(0\right)}\;,\\ \Delta \Psi_{2M,\;\pm ,\;↓}^{\left(0\right)}=\sum_{\begin{array}{l} l=1\\ l\neq 2M \end{array}}^{2M}\frac{\cos\frac{\theta_{l}^{\left(↓\right)}}{2}}{\sin h\frac{L\left(\gamma_{l}^{\left(↓\right)}-\gamma_{2M}^{\left(↓\right)}\right)}{2}}\Psi_{l,\;\pm }^{\left(0\right)}-\sum_{l=1}^{2M}\frac{i\sin\frac{\theta_{l}^{\left(↓\right)}}{2}}{\sinh\frac{L\left(\gamma_{l}^{\left(↓\right)}+\gamma_{M}^{\left(↓\right)}\right)}{2}}\Psi_{l,\;\mp }^{\left(0\right)}. \end{array}$$

In Table 1, (20) and (21), the values of $m^{\prime }$ and m are exactly the same as those in (7).

From Table 1, we see that all eigenvalues ${\left\{\;\tau^{\left(1\right)}\right\}}^{}$ of $H^{\left(0\right)}+\sinh2\phi_{1}H^{\left(1\right)}$ are nondegenerate. Hence, all degenerate eigenvalues of $H^{\left(0\right)}$ are relieved by $\sinh2\phi_{1}H^{\left(1\right)}$. Thus, when we calculate the eigenvalues and eigenvectors ${\left\{\;\tau^{\left(2\right)},\;\Psi^{\left(2\right)}\right\}}^{}$ of $H^{\left(0\right)}+\sinh2\phi_{1}H^{\left(1\right)}+\sinh2\phi_{2}H^{\left(2\right)}$, we only need use nondegenerate perturbation theory; this is applicable up to $\sinh2\phi_{Q}H^{\left(Q\right)}$. Further, since from ${\left\{\;\tau^{\left(q\right)},\;\Psi^{\left(q\right)}\right\}}^{}$ to ${\left\{\;\tau^{\left(q+1\right)},\;\Psi^{\left(q+1\right)}\right\}}^{}$ (q=1, 2, ⋯, Q−1), we need only to calculate the first-order approximation in terms of $\sinh2\phi_{q+1}\approx 2\phi_{q+1}$, and the corresponding recurrence formulas are:

(22) $$\begin{array}{l} \tau_{m}^{\left(q+1\right)}=\tau_{m}^{\left(q\right)}+\frac{\sinh2\phi_{q+1}}{2M}{\left(\Psi_{m}^{\left(q\right)}\right)}^{\;+}H^{\left(q+1\right)}\Psi_{m}^{\left(q\right)},\\ \Psi_{m}^{\left(q+1\right)}=\Psi_{m}^{\left(q\right)}-\frac{\sinh2\phi_{q+1}}{2M}\sum_{\begin{array}{l} \;l=1\\ l\neq m \end{array}}^{4M}\frac{{\left(\Psi_{l}^{\left(q\right)}\right)}^{+}H^{\left(q+1\right)}\Psi_{m}^{\left(q\right)}}{\tau_{l}^{\left(q\right)}-\tau_{m}^{\left(q\right)}}\Psi_{l}^{\left(q\right)}\\ \;(m=1,\;2,\;\cdots ,\;4M\;;\;q=1,\;2,\;\cdots ,\;Q-1)\;, \end{array},$$

In the calculation, all terms including the $\phi_{q}^{k}(k\geq 2)$ order are ignored.

In principle, by following the above approach we obtain the eigenvalues ${\left\{\;\tau^{\left(Q\right)}\right\}}^{}$. Furthermore, considering that up to the first-order approximation for $\phi_{q}$, we have $1+C\sinh2\phi_{q}\approx e^{C\sinh2\phi_{q}}$, the eigenvalues ${\left\{\;\tau^{\left(Q\right)}\right\}}^{}$ of H can be denoted by the forms:

$$e^{\pm \left(L\gamma_{m}^{\left(↑↓\right)}+\alpha_{m\;,\;↑↓}^{\left(Q\right)}\right)}{,\;e}^{\pm \left(L\gamma_{m}^{\left(↑↓\right)}-\beta_{m\;,\;↑↓}^{\left(Q\right)}\right)}{\;,\;e}^{\pm \left(L\gamma_{M}^{\left(↓\right)}+\delta_{M}^{\left(Q\right)}\right)}{,\;e}^{\pm \left(L\gamma_{2M}^{\left(↓\right)}+\delta_{2M}^{\left(Q\right)}\right)},$$

where the value of m is exactly the same as that in (7).

Based on the above forms of the eigenvalues ${\left\{\;\tau^{\left(Q\right)}\right\}}^{}$ and using the spinor analysis method, we obtain:

(23) $$\begin{array}{ll} {\tilde{Y}}_{Q} & =\left(\prod_{l=1}^{M}2\cosh\frac{L\gamma_{l}^{\left(↑\right)}+\alpha_{l\;,\;↑}^{\left(Q\right)}}{2}\right)\left(\prod_{m=1}^{M}2\cosh\frac{L\gamma_{m}^{\left(↑\right)}-\beta_{m\;,\;↑}^{\left(Q\right)}}{2}\right)\\ & +\left(\prod_{l=1}^{M}2\sinh\frac{L\gamma_{l}^{\left(↑\right)}+\alpha_{l\;,\;↑}^{\left(Q\right)}}{2}\right)\left(\prod_{m=1}^{M}2\sinh\frac{L\gamma_{m}^{\left(↑\right)}-\beta_{m\;,\;↑}^{\left(Q\right)}}{2}\right)\\ & +\left(2\cosh\frac{L\gamma_{2M}^{\left(↓\right)}+\delta_{2M}^{\left(Q\right)}}{2}\right)\left(2\cosh\frac{L\gamma_{M}^{\left(↓\right)}+\delta_{M}^{\left(Q\right)}}{2}\right)\left(\prod_{l=1}^{M-1}2\cosh\frac{L\gamma_{l}^{\left(↓\right)}+\alpha_{l\;,\;↓}^{\left(Q\right)}}{2}\right)\left(\prod_{m=1}^{M-1}2\cosh\frac{L\gamma_{m}^{\left(↓\right)}-\beta_{m\;,\;↓}^{\left(Q\right)}}{2}\right)\\ & +\left(2\sinh\frac{L\gamma_{2M}^{\left(↓\right)}+\delta_{2M}^{\left(Q\right)}}{2}\right)\left(2\sinh\frac{L\gamma_{M}^{\left(↓\right)}+\delta_{M}^{\left(Q\right)}}{2}\right)\left(\prod_{l=1}^{M-1}2\sinh\frac{L\gamma_{l}^{\left(↓\right)}+\alpha_{l\;,\;↓}^{\left(Q\right)}}{2}\right)\left(\prod_{m=1}^{M-1}2\sinh\frac{L\gamma_{m}^{\left(↓\right)}-\beta_{m\;,\;↓}^{\left(Q\right)}}{2}\right). \end{array}$$

Finally, according to (8) and (9), we obtain:

(24) $$\langle \sigma_{1,\;1}\sigma_{1,\;1+Q}\rangle =\frac{1}{\tilde{Z}}\left(\prod_{q=1}^{Q}\underset{\phi_{q}\to 0}{\lim}\frac{\partial }{\partial \phi_{q}}\right){\tilde{Y}}_{Q},$$

where $\tilde{Z}$ and ${\tilde{Y}}_{Q}$ are given by (6) and (23), respectively.

### 2.7. The Exact Expressions of $\langle \sigma_{1,\;1}\sigma_{1,\;2}\rangle$ and $\langle \sigma_{1,\;1}\sigma_{1,\;3}\rangle$ on a Finite Lattice

Although in Section 2.5 we presented a simplified approximate method, the actual calculation process of $\langle \sigma_{1,\;1}\sigma_{1,\;1+Q}\rangle$ is still complex; here, we only present the expressions of $\langle \sigma_{1,\;1}\sigma_{1,\;2}\rangle$ and $\langle \sigma_{1,\;1}\sigma_{1,\;3}\rangle$ directly.

When Q=1, substituting $\alpha_{m\;,\;↑↓}^{\left(1\right)}\;,\;\beta_{m\;,\;↑↓}^{\left(1\right)}\;,\;\delta_{M}^{\left(1\right)}$, and $\delta_{2M}^{\left(1\right)}$ given by (20) into (23), we obtain ${\tilde{Y}}_{1}$, and, further, we have:

(25) $$\begin{array}{ll} \underset{\phi_{1}\to 0}{\lim}\frac{\partial {\tilde{Y}}_{1}}{\partial \phi_{1}} & =\left(\sum_{m=1}^{M}\frac{\cos\theta_{m}^{\left(↑\right)}}{M}\tanh\frac{L\gamma_{m}^{\left(↑\right)}}{2}\right)\;{\left(\prod_{l=1}^{M}2\cosh\frac{L\gamma_{l}^{\left(↑\right)}}{2}\right)}^{2}\\ & +\left(\sum_{m=1}^{M}\frac{\cos\theta_{m}^{\left(↑\right)}}{M}\coth\frac{L\gamma_{m}^{\left(↑\right)}}{2}\right)\;{\left(\prod_{l=1}^{M}2\sinh\frac{L\gamma_{l}^{\left(↑\right)}}{2}\right)}^{2}\\ & +\left(\frac{1}{2M}\tanh\frac{L\gamma_{2M}^{\left(↓\right)}}{2}+\frac{1}{2M}\tanh\frac{L\gamma_{M}^{\left(↓\right)}}{2}+\sum_{m=1}^{M-1}\frac{\cos\theta_{m}^{\left(↓\right)}}{M}\tanh\frac{L\gamma_{m}^{\left(↓\right)}}{2}\right)\\ & \;\times \left(2\cosh\frac{L\gamma_{2M}^{\left(↓\right)}}{2}\right)\;\left(2\cosh\frac{L\gamma_{M}^{\left(↓\right)}}{2}\right)\;{\left(\prod_{l=1}^{M-1}2\cosh\frac{L\gamma_{l}^{\left(↓\right)}}{2}\right)}^{2}\\ & +\left(\frac{1}{2M}\coth\frac{L\gamma_{2M}^{\left(↓\right)}}{2}+\frac{1}{2M}\coth\frac{L\gamma_{M}^{\left(↓\right)}}{2}+\sum_{m=1}^{M-1}\frac{\cos\theta_{m}^{\left(↓\right)}}{M}\coth\frac{L\gamma_{m}^{\left(↓\right)}}{2}\right)\\ & \;\times \left(2\sinh\frac{L\gamma_{2M}^{\left(↓\right)}}{2}\right)\;\left(2\sinh\frac{L\gamma_{M}^{\left(↓\right)}}{2}\right)\;{\left(\prod_{l=1}^{M-1}2\sinh\frac{L\gamma_{l}^{\left(↓\right)}}{2}\right)}^{2}\;. \end{array}$$

Substituting $\tilde{Z}$ given by (6) and the above expression into (24), we obtain the expressions of $\langle \sigma_{1,\;1}\sigma_{1,\;2}\rangle$ of the model on a finite lattice:

(26) $$\langle \sigma_{1,\;1}\sigma_{1,\;2}\rangle =\frac{1}{\tilde{Z}}\underset{\phi_{1}\to 0}{\lim}\frac{\partial {\tilde{Y}}_{1}}{\partial \phi_{1}}.$$

Then, using ${\left\{\;\tau^{\left(1\right)},\;\Psi^{\left(1\right)}\right\}}^{}$ presented in Table 1, (20) and (21), and according to (22), we obtain ${\left\{\;\tau^{\left(2\right)}\right\}}^{}$, which can be denoted by the forms:

$$e^{\pm \left(L\gamma_{m}^{\left(↑↓\right)}+\alpha_{m\;,\;↑↓}^{\left(2\right)}\right)}{,\;e}^{\pm \left(L\gamma_{m}^{\left(↑↓\right)}-\beta_{m\;,\;↑↓}^{\left(2\right)}\right)}{\;,\;e}^{\pm \left(L\gamma_{M}^{\left(↓\right)}+\delta_{M}^{\left(2\right)}\right)}{,\;e}^{\pm \left(L\gamma_{2M}^{\left(↓\right)}+\delta_{2M}^{\left(2\right)}\right)},$$

where

$$\begin{array}{ll} \alpha_{m,\;↑↓}^{\left(2\right)} & =\frac{\sinh2\phi_{1}+\sinh2\phi_{2}}{M}\cos^{2}\frac{\theta_{m}}{2}-\frac{\sinh2\phi_{2}}{M}\sin^{2}\varphi_{m}\\ & +\frac{\sinh2\phi_{1}\sinh2\phi_{2}}{4M^{2}}\sum_{\begin{array}{c} k=1\\ k\neq m\;,\;k\neq m^{\prime } \end{array}}^{2M}\frac{{4\cos}^{2}\frac{\theta_{m}}{2}\cos^{2}\frac{\theta_{k}}{2}\cos\varphi_{m}\cos\varphi_{k}-\sin\theta_{m}\sin\theta_{k}\sin\varphi_{m}\sin\varphi_{k}}{e^{L\left(\gamma_{m}-\gamma_{k}\right)}-1}\\ & +\frac{\sinh2\phi_{1}\sinh2\phi_{2}}{4M^{2}}\sum_{k=1}^{2M}\frac{{4\cos}^{2}\frac{\theta_{m}}{2}\sin^{2}\frac{\theta_{k}}{2}\cos\varphi_{m}\cos\varphi_{k}+\sin\theta_{m}\sin\theta_{k}\sin\varphi_{m}\sin\varphi_{k}}{e^{L\left(\gamma_{m}+\gamma_{k}\right)}-1}\;, \end{array}$$

$$\begin{array}{ll} \beta_{m,\;↑↓}^{\left(2\right)} & =\frac{\sinh2\phi_{1}+\sinh2\phi_{2}}{M}\sin^{2}\frac{\theta_{m}}{2}-\frac{\sinh2\phi_{2}}{M}\sin^{2}\varphi_{m}\\ & +\frac{\sinh2\phi_{1}\sinh2\phi_{2}}{4M^{2}}\sum_{\begin{array}{c} k=1\\ k\neq m\;,\;k\neq m^{\prime } \end{array}}^{2M}\frac{-\;4\sin^{2}\frac{\theta_{m}}{2}\sin^{2}\frac{\theta_{k}}{2}\cos\varphi_{m}\cos\varphi_{k}+\;\sin\theta_{m}\sin\theta_{k}\sin\varphi_{m}\sin\varphi_{k}}{1-e^{-L\left(\gamma_{m}-\gamma_{k}\right)}}\\ & +\frac{\sinh2\phi_{1}\sinh2\phi_{2}}{4M^{2}}\sum_{k=1}^{2M}\frac{-4\sin^{2}\frac{\theta_{m}}{2}\cos^{2}\frac{\theta_{k}}{2}\cos\varphi_{m}\cos\varphi_{k}-\;\sin\theta_{m}\sin\theta_{k}\sin\varphi_{m}\sin\varphi_{k}}{1-e^{-L\left(\gamma_{m}+\gamma_{k}\right)}}\;, \end{array}$$

$$\begin{array}{ll} \delta_{M}^{\left(2\right)} & =\frac{\sinh2\phi_{1}+\sinh2\phi_{2}}{2M}-\frac{\sinh2\phi_{1}\sinh2\phi_{2}}{2M^{2}}\sum_{\begin{array}{c} \;k=1\\ k\neq M \end{array}}^{2M}\frac{1}{e^{L\left(\gamma_{M}^{\left(↓\right)}-\gamma_{k}^{\left(↓\right)}\right)}-1}\cos^{2}\frac{\theta_{k}^{\left(↓\right)}}{2}\cos\varphi_{k}^{\left(↓\right)}\\ & -\frac{\sinh2\phi_{1}\sinh2\phi_{2}}{2M^{2}}\sum_{k=1}^{2M}\frac{1}{e^{L\left(\gamma_{M}^{\left(↓\right)}+\gamma_{k}^{\left(↓\right)}\right)}-1}\sin^{2}\frac{\theta_{k}^{\left(↓\right)}}{2}\cos\varphi_{k}^{\left(↓\right)}, \end{array}$$

$$\begin{array}{ll} \delta_{2M}^{\left(2\right)} & =\frac{\sinh2\phi_{1}+\sinh2\phi_{2}}{2M}+\frac{\sinh2\phi_{1}\sinh2\phi_{2}}{2M^{2}}\sum_{k=1}^{2M-1}\frac{1}{e^{L\left(\gamma_{2M}^{\left(↓\right)}-\gamma_{k}^{\left(↓\right)}\right)}-1}\cos^{2}\frac{\theta_{k}^{\left(↓\right)}}{2}\cos\varphi_{k}^{\left(↓\right)}\\ & +\frac{\sinh2\phi_{1}\sinh2\phi_{2}}{2M^{2}}\sum_{k=1}^{2M}\frac{1}{e^{L\left(\gamma_{2M}^{\left(↓\right)}+\gamma_{k}^{\left(↓\right)}\right)}-1}\sin^{2}\frac{\theta_{k}^{\left(↓\right)}}{2}\cos\varphi_{k}^{\left(↓\right)}. \end{array}$$

In the above expressions, the values of $m^{\prime }$ and m are exactly the same as those in (7). We see that all terms with the factor $\sinh2\phi_{1}\sinh2\phi_{2}$ remain in the above expressions.

Substituting the above expressions of $\alpha_{m\;,\;↑↓}^{\left(2\right)}$, $\beta_{m\;,\;↑↓}^{\left(2\right)}$, $\delta_{M}^{\left(2\right)}$, and $\delta_{2M}^{\left(2\right)}$ into (23), we obtain ${\tilde{Y}}_{2}$, and, further, we have:

(27) $$\begin{array}{l} \underset{\phi_{2}\to 0}{\lim}\frac{\partial }{\partial \phi_{2}}\underset{\phi_{1}\to 0}{\lim}\frac{\partial {\tilde{Y}}_{2}}{\partial \phi_{1}}\\ =\left(\sum_{m=1}^{M}\frac{W_{m}^{\left(↑\right)}}{M}\tanh\frac{L\gamma_{m}^{\left(↑\right)}}{2}+\frac{1}{2M}\sum_{m=1}^{M}\frac{1}{M}\frac{2\cos^{2}\varphi_{m}^{\left(↑\right)}-\sin^{2}\theta_{m}^{\left(↑\right)}}{\cosh^{2}\frac{L\gamma_{m}^{\left(+\right)}}{2}}+{\left(\sum_{m=1}^{M}\frac{1}{M}\cos\theta_{m}^{\left(↑\right)}\tanh\frac{L\gamma_{m}^{\left(↑\right)}}{2}\right)}^{2}\right)\\ \;\times {\left(\prod_{l=1}^{M}2\cosh\frac{L\gamma_{l}^{\left(↑\right)}}{2}\right)}^{2}\\ +\left(\sum_{m=1}^{M}\frac{W_{m}^{\left(↑\right)}}{M}\coth\frac{L\gamma_{m}^{\left(↑\right)}}{2}-\frac{1}{2M}\sum_{m=1}^{M}\frac{1}{M}\frac{2\cos^{2}\varphi_{m}^{\left(↑\right)}-\sin^{2}\theta_{m}^{\left(↑\right)}}{\sinh^{2}\frac{L\gamma_{m}^{\left(+\right)}}{2}}+{\left(\sum_{m=1}^{M}\frac{1}{M}\cos\theta_{m}^{\left(↑\right)}\coth\frac{L\gamma_{m}^{\left(↑\right)}}{2}\right)}^{2}\right)\\ \;\times {\left(\prod_{l=1}^{M}2\sinh\frac{L\gamma_{l}^{\left(↑\right)}}{2}\right)}^{2}\\ +(\left(\frac{W_{2M}^{\left(↓\right)}}{2M}\tanh\frac{L\gamma_{2M}^{\left(↓\right)}}{2}+\frac{W_{M}^{\left(↓\right)}}{2M}\tanh\frac{L\gamma_{M}^{\left(↓\right)}}{2}+\sum_{m=1}^{M-1}\frac{W_{m}^{\left(↓\right)}}{M}\tanh\frac{L\gamma_{m}^{\left(↓\right)}}{2}\right)\\ \;+\frac{1}{2M}\left(\frac{1}{2M}\frac{1}{\cosh^{2}\frac{L\gamma_{2M}^{\left(↓\right)}}{2}}+\frac{1}{2M}\frac{1}{\cosh^{2}\frac{L\gamma_{M}^{\left(↓\right)}}{2}}+\sum_{m=1}^{M-1}\frac{1}{M}\frac{2\cos^{2}\varphi_{m}^{\left(↓\right)}-\sin^{2}\theta_{m}^{\left(↓\right)}}{\cosh^{2}\frac{L\gamma_{m}^{\left(↓\right)}}{2}}\right)\\ \;+{\left(\frac{1}{2M}\tanh\frac{L\gamma_{2M}^{\left(↓\right)}}{2}+\frac{1}{2M}\tanh\frac{L\gamma_{M}^{\left(↓\right)}}{2}+\sum_{m=1}^{M-1}\frac{1}{M}\cos\theta_{m}^{\left(↓\right)}\tanh\frac{L\gamma_{m}^{\left(↓\right)}}{2}\right)}^{2})\;\\ \;\times \left(2\cosh\frac{L\gamma_{2M}^{\left(↓\right)}}{2}\right)\left(2\cosh\frac{L\gamma_{M}^{\left(↓\right)}}{2}\right){\left(\prod_{l=1}^{M-1}2\cosh\frac{L\gamma_{l}^{\left(↓\right)}}{2}\right)}^{2}\\ +(\left(\frac{W_{2M}^{\left(↓\right)}}{2M}\coth\frac{L\gamma_{2M}^{\left(↓\right)}}{2}+\frac{W_{M}^{\left(↓\right)}}{2M}\coth\frac{L\gamma_{M}^{\left(↓\right)}}{2}+\sum_{m=1}^{M-1}\frac{W_{m}^{\left(↓\right)}}{M}\coth\frac{L\gamma_{m}^{\left(↓\right)}}{2}\right)\\ \;-\frac{1}{2M}\left(\frac{1}{2M}\frac{1}{\sinh^{2}\frac{L\gamma_{2M}^{\left(↓\right)}}{2}}+\frac{1}{2M}\frac{1}{\sinh^{2}\frac{L\gamma_{M}^{\left(↓\right)}}{2}}+\sum_{m=1}^{M-1}\frac{1}{M}\frac{2\cos^{2}\varphi_{m}^{\left(↓\right)}-\sin^{2}\theta_{m}^{\left(↓\right)}}{\sinh^{2}\frac{L\gamma_{m}^{\left(-\right)}}{2}}\right)\\ \;+{\left(\frac{1}{2M}\coth\frac{L\gamma_{2M}^{\left(↓\right)}}{2}+\frac{1}{2M}\coth\frac{L\gamma_{M}^{\left(↓\right)}}{2}+\sum_{m=1}^{M-1}\frac{1}{M}\cos\theta_{m}^{\left(↓\right)}\coth\frac{L\gamma_{m}^{\left(↓\right)}}{2}\right)}^{2})\\ \;\times \left(2\sinh\frac{L\gamma_{2M}^{\left(↓\right)}}{2}\right)\left(2\sinh\frac{L\gamma_{M}^{\left(↓\right)}}{2}\right){\left(\prod_{l=1}^{M-1}2\sinh\frac{L\gamma_{l}^{\left(↓\right)}}{2}\right)}^{2}\;, \end{array}$$

where $W_{m}^{\left(↑↓\right)}$ is introduced by:

(28) $$\begin{array}{l} W_{m}=\frac{1}{2M}(\sum_{\begin{array}{c} k=1\\ k\neq m\;,\;k\neq m^{\prime } \end{array}}^{2M}\frac{{4\cos}^{2}\frac{\theta_{m}}{2}\cos^{2}\frac{\theta_{k}}{2}\cos\varphi_{m}\cos\varphi_{k}-\sin\theta_{m}\sin\theta_{k}\sin\varphi_{m}\sin\varphi_{k}}{e^{L\left(\gamma_{m}-\gamma_{k}\right)}-1}\\ \;+\sum_{k=1}^{2M}\frac{{4\cos}^{2}\frac{\theta_{m}}{2}\sin^{2}\frac{\theta_{k}}{2}\cos\varphi_{m}\cos\varphi_{k}+\sin\theta_{m}\sin\theta_{k}\sin\varphi_{m}\sin\varphi_{k}}{e^{L\left(\gamma_{m}+\gamma_{k}\right)}-1}\\ \;+\sum_{\begin{array}{c} k=1\\ k\neq m\;,\;k\neq m^{\prime } \end{array}}^{2M}\frac{\;4\sin^{2}\frac{\theta_{m}}{2}\sin^{2}\frac{\theta_{k}}{2}\cos\varphi_{m}\cos\varphi_{k}-\;\sin\theta_{m}\sin\theta_{k}\sin\varphi_{m}\sin\varphi_{k}}{1-e^{-L\left(\gamma_{m}-\gamma_{k}\right)}}\\ \;+\sum_{k=1}^{2M}\frac{4\sin^{2}\frac{\theta_{m}}{2}\cos^{2}\frac{\theta_{k}}{2}\cos\varphi_{m}\cos\varphi_{k}+\;\sin\theta_{m}\sin\theta_{k}\sin\varphi_{m}\sin\varphi_{k}}{1-e^{-L\left(\gamma_{m}+\gamma_{k}\right)}})\;, \end{array}$$

where the value of $m^{\prime }$ is exactly the same as that in (7).

Substituting $\tilde{Z}$ given by (6) and (27) into (24), we obtain the expressions of $\langle \sigma_{1,\;1}\sigma_{1,\;3}\rangle$ of the model on a finite lattice:

(29) $$\langle \sigma_{1,\;1}\sigma_{1,\;3}\rangle =\frac{1}{\tilde{Z}}\underset{\phi_{2}\to 0}{\lim}\frac{\partial }{\partial \phi_{2}}\underset{\phi_{1}\to 0}{\lim}\frac{\partial {\tilde{Y}}_{2}}{\partial \phi_{1}}$$

### 2.8. The Expressions of $\langle \sigma_{1,\;1}\sigma_{1,\;2}\rangle$ and $\langle \sigma_{1,\;1}\sigma_{1,\;3}\rangle$ in the Thermodynamic Limit

We now consider the thermodynamic limit. First, if L is very large, then according to (7) we have:

$$2\cosh\frac{L\gamma_{m}^{\left(↑↓\right)}}{2}\approx 2\sinh\frac{L\gamma_{m}^{\left(↑↓\right)}}{2}\approx \exp\frac{L\gamma_{m}^{\left(↑↓\right)}}{2},\tanh\frac{L\gamma_{m}^{\left(↑↓\right)}}{2}\approx \coth\frac{L\gamma_{m}^{\left(↑↓\right)}}{2}\approx 1\;(m=1,2\cdots M);$$

However, when the system crosses its critical temperature, $\gamma_{2M}^{\left(↓\right)}=2(K-K^{\prime })$ changes sign, following which we therefore have:

$$2\cosh\frac{L\gamma_{2M}^{\left(↓\right)}}{2}\approx \exp\frac{L\left|\gamma_{2M}^{\left(↓\right)}\right|}{2},\;2\sinh\frac{L\gamma_{2M}^{\left(↓\right)}}{2}\approx \frac{\gamma_{2M}^{\left(↓\right)}}{\left|\gamma_{2M}^{\left(↓\right)}\right|}\exp\frac{L\left|\gamma_{2M}^{\left(↓\right)}\right|}{2},\tanh\frac{L\gamma_{2M}^{\left(↓\right)}}{2}\approx \coth\frac{L\gamma_{2M}^{\left(↓\right)}}{2}\approx \frac{\gamma_{2M}^{\left(↓\right)}}{\left|\gamma_{2M}^{\left(-\right)}\right|}.$$

Hence, for $\tilde{Z}$ and $\underset{\phi_{1}\to 0}{\lim}\frac{\partial {\tilde{Y}}_{1}}{\partial \phi_{1}}$ given by (6) and (25), respectively, when L is very large, we obtain:

(30) $$\begin{array}{l} \tilde{Z}\approx \frac{1}{2}({\left(\prod_{m=1}^{M}\exp\frac{L\gamma_{m}^{\left(↑\right)}}{2}\right)}^{2}+{\left(\prod_{m=1}^{M}\exp\frac{L\gamma_{m}^{\left(↑\right)}}{2}\right)}^{2}\\ \;+\exp\frac{L\left|\gamma_{2M}^{\left(↓\right)}\right|}{2}\exp\frac{L\gamma_{M}^{\left(↓\right)}}{2}{\left(\prod_{m=1}^{M-1}\exp\frac{L\gamma_{m}^{\left(↓\right)}}{2}\right)}^{2}+\frac{\gamma_{2M}^{\left(↓\right)}}{\left|\gamma_{2M}^{\left(↓\right)}\right|}\exp\frac{L\left|\gamma_{2M}^{\left(↓\right)}\right|}{2}\exp\frac{L\gamma_{M}^{\left(↓\right)}}{2}{\left(\prod_{m=1}^{M-1}\exp\frac{L\gamma_{m}^{\left(↓\right)}}{2}\right)}^{2})\\ \approx {\left(\prod_{m=1}^{M}\exp\frac{L\gamma_{m}^{\left(↑\right)}}{2}\right)}^{2}\times \left\{ \begin{array}{l} 1\;,\;K<K^{\prime }\;;\\ 2\;,\;K>K^{\prime }\;, \end{array}\right. \end{array}$$

$$\begin{array}{l} \underset{\phi_{1}\to 0}{\lim}\frac{\partial {\tilde{Y}}_{1}}{\partial \phi_{1}}\approx \left(\sum_{n=1}^{M}\frac{\cos\theta_{n}^{\left(↑\right)}}{M}\right)\;{\left(\prod_{l=1}^{M}\exp\frac{L\gamma_{l}^{\left(↑\right)}}{2}\right)}^{2}\\ \;+\frac{1}{2}\left(\frac{1}{2M}\frac{\gamma_{2M}^{\left(↓\right)}}{\left|\gamma_{2M}^{\left(-\right)}\right|}+\frac{1}{2M}+\sum_{n=1}^{M-1}\frac{\cos\theta_{n}^{\left(↓\right)}}{M}\right)\;\left(1+\frac{\gamma_{2M}^{\left(↓\right)}}{\left|\gamma_{2M}^{\left(-\right)}\right|}\right)\;\left(\exp\frac{L\left|\gamma_{2M}^{\left(↓\right)}\right|}{2}\right)\;\left(\exp\frac{L\gamma_{M}^{\left(↓\right)}}{2}\right)\;{\left(\prod_{l=1}^{M-1}\exp\frac{L\gamma_{l}^{\left(↓\right)}}{2}\right)}^{2}\\ \approx \left(\sum_{n=1}^{M}\frac{\cos\theta_{n}^{\left(↑\right)}}{M}\right)\;{\left(\prod_{m=1}^{M}\exp\frac{L\gamma_{m}^{\left(↑\right)}}{2}\right)}^{2}\times \left\{ \begin{array}{l} 1\;,\;K<K^{\prime }\;;\\ 2\;,\;K>K^{\prime }\;. \end{array}\right. \end{array}$$

Substituting the above two expressions into (26), we obtain:

(31) $$\begin{array}{l} \underset{\begin{array}{l} L\to \infty \\ N\to \infty \end{array}}{\lim}\langle \sigma_{1\;,\;1}\sigma_{1\;,\;2}\rangle =\underset{M\to \infty }{\lim}\sum_{m=1}^{M}\frac{\cos\theta_{m}^{\left(↑\right)}}{M}=\underset{M\to \infty }{\lim}\left(\frac{1}{2}\sum_{m=1}^{M}\frac{\cos\theta_{m}^{\left(↑\right)}}{M}+\frac{1}{2}\sum_{m=M+1}^{2M}\frac{\cos\theta_{m}^{\left(↑\right)}}{M}\right)\\ =\underset{N\to \infty }{\lim}\sum_{n=1}^{N}\frac{\cos\theta_{n}^{\left(↑\right)}}{N}=\int_{0}^{1}dx\cos\theta (\pi x)\;, \end{array}$$

where the function θ(πx) in terms of (14) is defined by:

(32) $$\begin{array}{l} \cos\theta (\pi x)=\frac{\cosh2K^{\prime }\sinh2K-\cos(\pi x)\sinh2K^{\prime }\cosh2K}{\sqrt{{\left(\cosh2K^{\prime }\cosh2K-\cos(\pi x)\sinh2K^{\prime }\sinh2K\right)}^{2}-1}}\;,\\ \sin\theta (\pi x)=\frac{\sin(\pi x)\sinh2K^{\prime }}{\sqrt{{\left(\cosh2K^{\prime }\cosh2K-\cos(\pi x)\sinh2K^{\prime }\sinh2K\right)}^{2}-1}}\;. \end{array}$$

The result (31) is in accordance with that in Reference [8].

According to the similar discussions, for $\langle \sigma_{1,\;1}\sigma_{1,\;3}\rangle$ we first have:

(33) $$\underset{\begin{array}{l} L\to \infty \\ N\to \infty \end{array}}{\lim}\langle \sigma_{1,\;1}\sigma_{1,\;3}\rangle \approx \underset{\begin{array}{l} L\to \infty \\ M\to \infty \end{array}}{\lim}\sum_{m=1}^{M}\frac{W_{m}^{\left(↑\right)}}{M}+{\left(\underset{M\to \infty }{\lim}\sum_{m=1}^{M}\frac{\cos\theta_{m}^{\left(↑\right)}}{M}\right)}^{2}.$$

We discuss the first term in (28) as an example to show how to calculate $\underset{L\to \infty }{\lim}W_{m}^{\left(+\right)}$. First, using (7) and (15), the first term in (28) can be written in the form:

$$\begin{array}{l} \frac{1}{2M}\sum_{\begin{array}{c} k=1\\ k\neq m\;,\;k\neq m^{\prime } \end{array}}^{2M}\frac{{4\cos}^{2}\frac{\theta_{m}^{\left(↑\right)}}{2}\cos^{2}\frac{\theta_{k}^{\left(↑\right)}}{2}\cos\varphi_{m}^{\left(↑\right)}\cos\varphi_{k}^{\left(↑\right)}-\sin\theta_{m}^{\left(↑\right)}\sin\theta_{k}^{\left(↑\right)}\sin\varphi_{m}^{\left(↑\right)}\sin\varphi_{k}^{\left(↑\right)}}{e^{L\left(\gamma_{m}^{\left(↑\right)}-\gamma_{k}^{\left(↑\right)}\right)}-1}\\ =\frac{1}{M}\sum_{\begin{array}{c} \;k=1\\ k\neq m \end{array}}^{M}\frac{{4\cos}^{2}\frac{\theta_{m}^{\left(↑\right)}}{2}\cos^{2}\frac{\theta_{k}^{\left(↑\right)}}{2}\cos\varphi_{m}^{\left(↑\right)}\cos\varphi_{k}^{\left(↑\right)}-\sin\theta_{m}^{\left(↑\right)}\sin\theta_{k}^{\left(↑\right)}\sin\varphi_{m}^{\left(↑\right)}\sin\varphi_{k}^{\left(↑\right)}}{e^{L\left(\gamma_{m}^{\left(↑\right)}-\gamma_{k}^{\left(↑\right)}\right)}-1}\\ =\frac{1}{M}\sum_{k=1}^{m-1}\frac{{4\cos}^{2}\frac{\theta_{m}^{\left(↑\right)}}{2}\cos^{2}\frac{\theta_{k}^{\left(↑\right)}}{2}\cos\varphi_{m}^{\left(↑\right)}\cos\varphi_{k}^{\left(↑\right)}-\sin\theta_{m}^{\left(↑\right)}\sin\theta_{k}^{\left(↑\right)}\sin\varphi_{m}^{\left(↑\right)}\sin\varphi_{k}^{\left(↑\right)}}{e^{L\left(\gamma_{m}^{\left(↑\right)}-\gamma_{k}^{\left(↑\right)}\right)}-1}\\ +\frac{1}{M}\sum_{k=m+1}^{M}\frac{{4\cos}^{2}\frac{\theta_{m}^{\left(↑\right)}}{2}\cos^{2}\frac{\theta_{k}^{\left(↑\right)}}{2}\cos\varphi_{m}^{\left(↑\right)}\cos\varphi_{k}^{\left(↑\right)}-\sin\theta_{m}^{\left(↑\right)}\sin\theta_{k}^{\left(↑\right)}\sin\varphi_{m}^{\left(↑\right)}\sin\varphi_{k}^{\left(↑\right)}}{e^{L\left(\gamma_{m}^{\left(↑\right)}-\gamma_{k}^{\left(↑\right)}\right)}-1}\;. \end{array}$$

According to (7), when k<m, $\gamma_{k}^{\left(↑\right)}<\gamma_{m}^{\left(↑\right)}$, $\underset{L\to \infty }{\lim}e^{L\left(\gamma_{m}^{\left(↑\right)}-\gamma_{k}^{\left(↑\right)}\right)}=\infty$, and, thus, the first term in the above expression vanishes; when k>m, $\gamma_{k}^{\left(↑\right)}>\gamma_{m}^{\left(↑\right)}$, $\underset{L\to \infty }{\lim}e^{L\left(\gamma_{m}^{\left(↑\right)}-\gamma_{k}^{\left(↑\right)}\right)}=0$, we therefore obtain:

$$\begin{array}{l} \underset{L\to \infty }{\lim}\frac{1}{2M}\sum_{\begin{array}{c} k=1\\ k\neq m\;,\;k\neq m^{\prime } \end{array}}^{2M}\frac{{4\cos}^{2}\frac{\theta_{m}^{\left(↑\right)}}{2}\cos^{2}\frac{\theta_{k}^{\left(↑\right)}}{2}\cos\varphi_{m}^{\left(↑\right)}\cos\varphi_{k}^{\left(↑\right)}-\sin\theta_{m}^{\left(↑\right)}\sin\theta_{k}^{\left(↑\right)}\sin\varphi_{m}^{\left(↑\right)}\sin\varphi_{k}^{\left(↑\right)}}{e^{L\left(\gamma_{m}^{\left(↑\right)}-\gamma_{k}^{\left(↑\right)}\right)}-1}\\ =\frac{1}{M}\sum_{k=m+1}^{M}\frac{{4\cos}^{2}\frac{\theta_{m}^{\left(↑\right)}}{2}\cos^{2}\frac{\theta_{k}^{\left(↑\right)}}{2}\cos\varphi_{m}^{\left(↑\right)}\cos\varphi_{k}^{\left(↑\right)}-\sin\theta_{m}^{\left(↑\right)}\sin\theta_{k}^{\left(↑\right)}\sin\varphi_{m}^{\left(↑\right)}\sin\varphi_{k}^{\left(↑\right)}}{-1}\;. \end{array}$$

Using this method to deal with the remaining terms in $W_{m}^{\left(↑\right)}$, we finally obtain:

$$\begin{array}{l} \underset{L\to \infty }{\lim}W_{m}^{\left(↑\right)}=\frac{2}{M}(\sum_{k=m+1}^{M}\sin\theta_{m}^{\left(↑\right)}\sin\theta_{k}^{\left(↑\right)}\sin\varphi_{m}^{\left(↑\right)}\sin\varphi_{k}^{\left(↑\right)}-\sum_{k=m+1}^{M}\cos\theta_{m}^{\left(↑\right)}\cos\theta_{k}^{\left(↑\right)}\cos\varphi_{m}^{\left(↑\right)}\cos\varphi_{k}^{\left(↑\right)}\\ \;+\sum_{k=1}^{m+1}\cos\varphi_{m}^{\left(↑\right)}\cos\varphi_{k}^{\left(↑\right)})+\frac{2}{M}\left(2\sin^{2}{\frac{\theta_{m}^{\left(↑\right)}}{2}}^{2}\left(\cos^{2}\frac{\theta_{m}^{\left(↑\right)}}{2}-\cos^{2}\varphi_{m}^{\left(↑\right)}\right)-\cos\varphi_{m}^{\left(↑\right)}\cos\varphi_{m+1}^{\left(↑\right)}\right). \end{array}$$

As M→∞, the second term in the above expression vanishes, and, according to the definition of the Riemann integral, we have:

$$\begin{array}{ll} \underset{\begin{array}{l} L\to \infty \\ M\to \infty \end{array}}{\lim}W_{m}^{\left(↑\right)} & =2\int_{x}^{1}dy\left(\sin\theta (\pi x)\sin\theta (\pi y)\sin(\pi x)\sin(\pi y)-\cos\theta (\pi x)\cos\theta (\pi y)\cos(\pi x)\cos(\pi y)\right)\\ & +2\int_{0}^{x}dy\;\cos(\pi x)\cos(\pi y)\;, \end{array}$$

where the function θ(πx) is introduced by (32), $x=\frac{m+1}{M}$. Further,

(34) $$\begin{array}{ll} \underset{\begin{array}{l} L\to \infty \\ M\to \infty \end{array}}{\lim}\sum_{m=1}^{M}\frac{W_{m}^{\left(↑\right)}}{M} & =2\int_{0}^{1}dx\int_{x}^{1}dy\sin\theta (\pi x)\sin\theta (\pi y)\sin(\pi x)\sin(\pi y)\\ & -2\int_{0}^{1}dx\int_{x}^{1}dy\cos\theta (\pi x)\cos\theta (\pi y)\cos(\pi x)\cos(\pi y)+2\int_{0}^{1}dx\int_{0}^{x}dy\;\cos(\pi x)\cos(\pi y)\;. \end{array}$$

**Figure 1:**
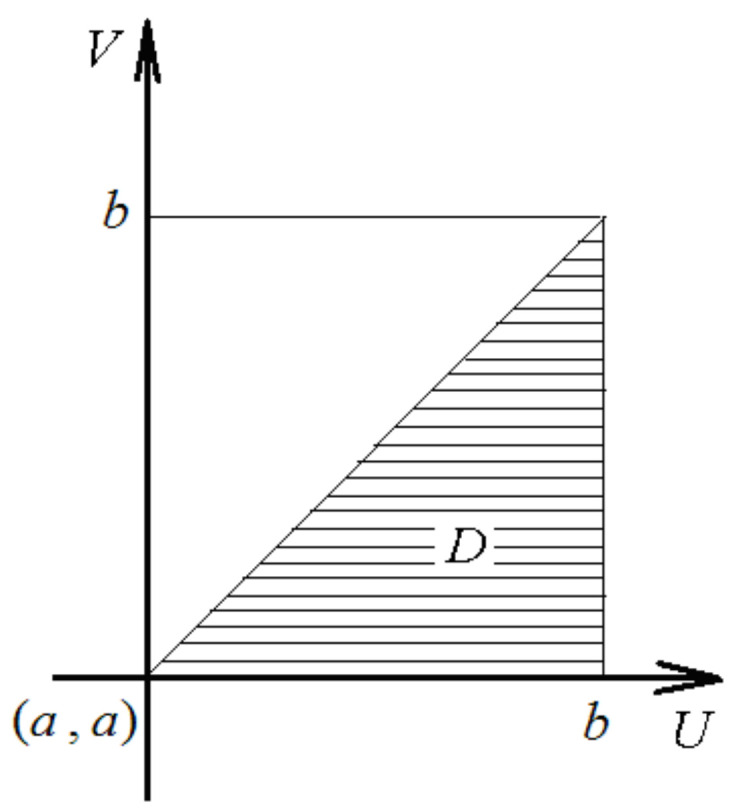
The domain of the integration in (35).

Generally speaking, for the function f(u, v) and the domain D of the integration shown in Figure 1, we have:

(35) $$\iint_{D}dudvf(u,\;v)=\int_{a}^{b}\;du\int_{a}^{\;u}\;dvf(u,\;v)=\int_{a}^{b}dv\int_{v}^{b}\;duf(u,\;v)\;.$$

Using (35), for the first term in (34) we obtain:

$$\begin{array}{l} 2\int_{0}^{1}dx\int_{x}^{1}dy\;\sin\theta (\pi x)\sin\theta (\pi y)\sin(\pi x)\sin(\pi y)=2\int_{0}^{1}dx\sin\theta (\pi x)\sin(\pi x)\int_{x}^{1}dy\;\sin\theta (\pi y)\sin(\pi y)\\ =\int_{0}^{1}dx\;\sin\theta (\pi x)\sin(\pi x)\int_{x}^{1}dy\;\sin\theta (\pi y)\sin(\pi y)+\int_{0}^{1}dy\;\sin\theta (\pi y)\sin(\pi y)\int_{0}^{y}dx\;\sin\theta (\pi x)\sin(\pi x)\\ =\int_{0}^{1}dx\;\sin\theta (\pi x)\sin(\pi x)\int_{0}^{1}dy\;\sin\theta (\pi y)\sin(\pi y)\;. \end{array}$$

Likewise, the second term in (34) becomes:

$$-2\int_{0}^{1}dx\int_{x}^{1}dy\;\cos\theta (\pi x)\cos\theta (\pi y)\cos(\pi x)\cos(\pi y)=-\int_{0}^{1}dx\;\cos\theta (\pi x)\cos(\pi x)\int_{0}^{1}dy\;\cos\theta (\pi y)\cos(\pi y)\;.$$

Further, the third term in (34) vanishes due to:

$$\int_{0}^{1}dx\int_{0}^{x}dy\;\cos(\pi x)\cos(\pi y)=\frac{1}{\pi }\int_{0}^{1}dx\cos(\pi x)\sin(\pi x)=0$$

Therefore, (34) becomes:

(36) $$\begin{array}{l} \underset{\begin{array}{l} L\to \infty \\ M\to \infty \end{array}}{\lim}\sum_{m=1}^{M}\frac{W_{m}^{\left(↑\right)}}{M}=\int_{0}^{1}dx\;\sin\theta (\pi x)\sin(\pi x)\int_{0}^{1}dy\;\sin\theta (\pi y)\sin(\pi y)\\ \;-\int_{0}^{1}dx\;\cos\theta (\pi x)\cos(\pi x)\int_{0}^{1}dy\;\cos\theta (\pi y)\cos(\pi y)\\ =-\int_{0}^{1}dx\;\cos(\theta (\pi x)-\pi x)\int_{0}^{1}dy\;\cos(\theta (\pi y)+\pi y)\;. \end{array}$$

Substituting (31) and (36) into (33), we obtain the form of $\langle \sigma_{1,\;1}\sigma_{1,\;3}\rangle$ in the thermodynamic limit:

(37) $$\underset{\begin{array}{l} L\to \infty \\ N\to \infty \end{array}}{\lim}\langle \sigma_{1,\;1}\sigma_{1,\;3}\rangle =-\int_{0}^{1}dx\;\cos(\theta (\pi x)-\pi x)\int_{0}^{1}dy\;\cos(\theta (\pi y)+\pi y)+{\left(\int_{0}^{1}dx\cos\theta (\pi x)\right)}^{2}\;.$$

On the other hand, the expressions of $\langle \sigma_{1,\;1}\sigma_{1,\;1+Q}\rangle$ in the thermodynamic limit have been obtained [3,5,9]. Thus, we here cite the formulas (B6) and (B7) in Reference [9] for comparison. According to those two formulas:

(38) $$\underset{\begin{array}{l} L\to \infty \\ N\to \infty \end{array}}{\lim}\langle \sigma_{1\;,\;1}\sigma_{1\;,\;2}\rangle =a_{0},\underset{\begin{array}{l} L\to \infty \\ N\to \infty \end{array}}{\lim}\langle \sigma_{1\;,\;1}\sigma_{1\;,\;3}\rangle =\left|\begin{array}{cc} a_{0} & a_{1}\\ a_{-1} & a_{0} \end{array}\right|=a_{0}^{2}-a_{1}a_{-1};a_{r}=\frac{1}{\pi }\int_{0}^{\pi }\;d\omega \cos(\theta (\omega )-r\omega ).$$

where θ(ω) is the function δ∗(ω) in Reference [9]. We see that (31) and (37) obtained here are exactly the same as (38).

## 3. Long Range-Order in the Model with Periodic-Free Boundary Conditions

For the model with L rows and N columns and periodic boundary condition in the horizontal direction and free boundary condition in the vertical direction, we consider $\langle \sigma_{l\;,\;n}\sigma_{l\;,\;n^{\prime }}\rangle$, i.e., correlation functions of pairwise spins in one column, periodic boundary condition in the horizontal direction leads to:

(39) $$\langle \sigma_{l\;,\;n}\sigma_{l\;,\;n^{\prime }}\rangle =\langle \sigma_{1\;,\;n}\sigma_{1\;,\;n^{\prime }}\rangle =\frac{1}{Z_{0}}\sum_{\left\{\sigma_{h\;,\;v}=\pm 1\right\}}\sigma_{1\;,\;1}\sigma_{1\;,\;n^{\prime }}\exp\left(\frac{J^{\prime }}{kT}\sum_{l=1}^{L}\sum_{m=1}^{N}\sigma_{l\;,\;m}\sigma_{l+1\;,\;m}\right)\exp\left(\frac{J}{kT}\sum_{l=1}^{L}\sum_{m=1}^{N-1}\sigma_{l\;,\;m}\sigma_{l\;,\;m+1}\right)\;,$$

where

(40) $$Z_{0}=\sum_{\left\{\sigma_{h\;,\;v}=\pm 1\right\}}\exp\left(\frac{J^{\prime }}{kT}\sum_{l=1}^{L}\sum_{m=1}^{N}\sigma_{l\;,\;m}\sigma_{l+1\;,\;m}\right)\exp\left(\frac{J}{kT}\sum_{l=1}^{L}\sum_{m=1}^{N-1}\sigma_{l\;,\;m}\sigma_{l\;,\;m+1}\right)$$

is the partition function of the system in absence of a magnetic field, where $\sigma_{L+1\;,\;m}=\sigma_{1\;,\;m}$, $J^{\prime }(>0)$ and J(>0) are the interaction constants for the horizontal and vertical directions, respectively.

### 3.1. Some Results Concerning the Partition Function $Z_{0}$

We summarize some results concerning $Z_{0}$ given by (40), some of which are obtained in Reference [12]. However, the approximate values of some quantities presented here show improvement over those given by Reference [12].

By using the spinor analysis method, $Z_{0}$ is obtained in Reference [12]:

(41) $$Z_{0}={\left(2\sinh\frac{2J^{\prime }}{kT}\right)}^{\frac{LN}{2}}\prod_{n=1}^{N}\left(2\cosh\frac{L\gamma_{n-1}}{2}\right)\;,$$

where $\gamma_{n-1}$ (n=1, 2, ⋯, N) are determined by:

(42) $$\cosh\gamma_{n-1}=\cosh2K^{\prime }\cosh2K-x_{n-1}\sinh2K^{\prime }\sinh2K,e^{-2K^{\prime }}=\tanh\frac{J^{\prime }}{kT}\;,\;K=\frac{J}{kT},$$

where (n=2, 3, ⋯, N) (n=1, 2, ⋯, N) are *N* roots of the *N*-th order algebraic equation in *x*:

(43) $$g_{N}(x)-2g_{N-1}(x)\coth2K^{\prime }\tanh K+g_{N-2}(x)\tanh^{2}K=0,$$

where

(44) $$g_{n}(x)=\sum_{k=0}^{\left[n/2\right]}\frac{(n+1)!}{(2k+1)!(n-2k)!}x^{n-2k}{\left(x^{2}-1\right)}^{\;k}$$

is an *n*-th degree polynomial in  x. If by $x\equiv \frac{d+d^{-1}}{2}$ we introduce the quantity d, then $g_{n}(x)$ can be written in the form:

(45) $$g_{n}(x)=\frac{d^{n+1}-d^{-(n+1)}}{d-d^{-1}}.$$

The expression in (45) is not only simple but also convenient for investigating the properties of $g_{n}(x)$, especially if we assume x=cosφ, then $g_{n}(x)=\frac{\sin(n+1)\varphi }{\sin\varphi }$. Substituting these forms of $g_{n}(x)$ into (43), for the N−1 roots of the *N*-th order algebraic Equation (43) we obtain:

(46) $$x_{n-1}=\cos\varphi_{n-1},\varphi_{n-1}=\frac{(n-1)\pi +\theta_{n-1}}{N},0<\theta_{n-1}<\pi \left(n=2,\;3,\;\cdots ,\;N\right),$$

Further, $\theta_{n-1}$ can be determined by solving a transcendental equation about θ; if *N* is finite, then the evaluation of $\theta_{n-1}$ is complex because of the so-called “finite size effect”; for the limit case $\langle \sigma_{l,\;1}\sigma_{l,\;N}\rangle$, we can assume $\theta_{n-1}=\sum_{k=0}^{\infty }\frac{\theta_{n-1}^{\left(k\right)}}{N^{k}}$ and obtain $\theta_{n-1}$ by the iterative method. Further, we obtain $\gamma_{1}\;,\;\gamma_{2}\;,\;\cdots \;,\;\gamma_{N-1}$ in terms of (42). Concretely, correcting to $\frac{1}{N}$ order, we have:

(47) $$\begin{array}{c} x_{n-1}=\cos\frac{(n-1)\pi +\theta_{n-1}^{\left(0\right)}}{N}\;,\;\gamma_{n-1}\approx \gamma_{n-1}^{\left(0\right)}+2\sin\frac{(n-1)\pi +\theta_{n-1}^{\left(0\right)}}{N}\sin\frac{\theta_{n-1}^{\left(0\right)}}{2N}\frac{\sinh2K^{\prime }\sinh2K}{\sinh\gamma_{n-1}^{\left(0\right)}}\;,\\ \left(n=2,\;3,\;\cdots ,\;N\right) \end{array}$$

where $\gamma_{n-1}^{\left(0\right)}$ and $\theta_{n-1}^{\left(0\right)}$ (n=2, 3, ⋯, N) are introduced by:

(48) $$\begin{array}{l} \cosh\gamma_{n-1}^{\left(0\right)}=\cosh2K^{\prime }\cosh2K-\cos\frac{(n-1)\pi }{N}\sinh2K^{\prime }\sinh2K\;,\;\gamma_{n-1}^{\left(0\right)}>0;\\ \cosh2K^{\prime }\sinh2K-\cos\frac{(n-1)\pi }{N}\sinh2K^{\prime }\cosh2K=\sinh\gamma_{n-1}^{\left(0\right)}\cos\theta_{n-1}^{\left(0\right)}\;,\\ \sin\frac{(n-1)\pi }{N}\sinh2K^{\prime }=\sinh\gamma_{n-1}^{\left(0\right)}\sin\theta_{k}^{\left(0\right)}\;,\;0<\theta_{n-1}^{\left(0\right)}<\pi \;. \end{array}$$

More important are the values of $x_{0}$ and $\gamma_{0}$; to present $x_{0}$ and $\gamma_{0}$, we first introduce a temperature $T_{c}$ in terms of:

(49) $$N=\frac{\sinh2{K^{\prime }}_{c}}{\sinh2(K_{c}-{K^{\prime }}_{c})},\;\tanh{K^{\prime }}_{c}=e^{-\frac{2J^{\prime }}{kT_{c}}},\;K_{c}=\frac{J}{kT_{c}}.$$

When $T\geq T_{c}$, $0<x_{0}\leq 1$; however, when $T<T_{c}$, $K^{\prime }<K$ and $1<x_{0}<\frac{\tanh2K}{\tanh2K^{\prime }}$. For the limit case N→∞, we can obtain the approximate values of $x_{0}$ and $\gamma_{0}$, whose low-order approximations are:

(50) $$x_{0}\approx \left\{ \begin{array}{ll} \cos\frac{\pi }{N}, & T\geq T_{c},\;K^{\prime }>K\;;\\ \cos\frac{\pi }{2N}, & T\geq T_{c},\;K^{\prime }=K\;;\\ \frac{1}{2}\left(\frac{\tanh K}{\tanh K^{\prime }}+\frac{\tanh K^{\prime }}{\tanh K}\right)-2{\left(\frac{\tanh K^{\prime }}{\tanh K}\right)}^{2N}\frac{{\left(\cosh2K-\cosh2K^{\prime }\right)}^{2}}{\sin h2K^{\prime }\sinh^{3}2K}, & T<T_{c}, \end{array}\right.$$

(51) $$\gamma_{0}\approx \left\{ \begin{array}{ll} 2(K^{\prime }-K)+2\sin^{2}\frac{\pi }{2N}\frac{\sinh2K^{\prime }\sinh2K}{\sinh2(K^{\prime }-K)}, & T\geq T_{c},\;K^{\prime }>K\;;\\ 2\sin\frac{\pi }{4N}\sinh2K, & T\geq T_{c},\;K^{\prime }=K\;;\\ 2{\left(\frac{\tanh K^{\prime }}{\tanh K}\right)}^{N}\frac{\cosh2K-\cosh2K^{\prime }}{\sinh2K}, & T<T_{c}\;. \end{array}\right.$$

We can thus make a comparison between Onsager’s lattice and the model with periodic-free boundary conditions. For Onsager’s lattice, when the system crosses its critical temperature, $\gamma_{N}^{\left(-\right)}=\gamma_{2M}^{\left(-\right)}=2(K-K^{\prime })$ given by (7) changes sign; however, from (51) we see that, for the model with periodic-free boundary conditions, when $T\geq T_{c}$, $\gamma_{0}\approx 2(K^{\prime }-K)$. Once the system crosses its critical temperature $T_{c}$, $\gamma_{0}$ becomes exponentially smaller and then vanishes rapidly as N→∞. This property of $\gamma_{0}$ plays a key role for the correlation function $\langle \sigma_{1,\;1}\sigma_{1,\;N}\rangle$.

### 3.2. The Matrix Forms of $\langle \sigma_{1,\;1}\sigma_{1,\;N-Q}\rangle$ and Some Results Concerning $\langle \sigma_{1,\;1}\sigma_{1,\;N}\rangle$ Obtained in Reference [12]

If we write (39) in forms similar to (8), then by employing the method presented in Section 2, the exact expressions we can obtain are still $\langle \sigma_{1,\;1}\sigma_{1,\;2}\rangle \;,$ $\langle \sigma_{1,\;1}\sigma_{1,\;3}\rangle \;,\;\cdots$, which belong to the short-range order. We still cannot obtain the exact expressions of $\langle \sigma_{l,\;1}\sigma_{l,\;N}\rangle$, $\langle \sigma_{l,\;1}\sigma_{l,\;N-1}\rangle \;,\;\cdots$.

To obtain the exact expressions of $\langle \sigma_{l,\;1}\sigma_{l,\;N}\rangle$, $\langle \sigma_{l,\;1}\sigma_{l,\;N-1}\rangle \;,\;\cdots$, we consider the forms:

(52) $$\langle \sigma_{1,\;1}\sigma_{1,\;N-n}\rangle =\frac{1}{Z_{0}}\sum_{\left\{\sigma_{h\;,\;v}=\pm 1\right\}}\sigma_{1,\;1}\sigma_{1,\;N-n}\exp\left(\frac{J^{\prime }}{kT}\sum_{l=1}^{L}\sum_{m=1}^{N}\sigma_{l\;,\;m}\sigma_{l+1\;,\;m}\right)\exp\left(\frac{J}{kT}\sum_{l=1}^{L}\sum_{m=1}^{N-1}\sigma_{l\;,\;m}\sigma_{l\;,\;m+1}\right).$$

Taking advantage of $\sigma_{l\;,\;m}^{2}=1$ and $\sigma_{1\;,\;l}\sigma_{1\;,\;m}=\underset{\alpha \to 0}{\lim}\frac{\partial e^{\alpha \sigma_{1\;,\;l}\sigma_{1\;,\;m}}}{\partial \alpha }$, we have:

$$\begin{array}{l} \sigma_{1,\;1}\sigma_{1,\;N-n}=\sigma_{1,\;1}\sigma_{1,\;N}\sigma_{1,\;N}\sigma_{1,\;N-1}\cdots \sigma_{1,\;N-(n-2)}\sigma_{1,\;N-(n-1)}\sigma_{1,\;N-(n-1)}\sigma_{1,\;N-n}\\ =\left(\prod_{k=1}^{n}\underset{\beta_{k}\to 0}{\lim}\frac{\partial }{\partial \beta_{k}}\right)\underset{\beta_{N}\to 0}{\lim}\frac{\partial }{\partial \beta_{N}}\left(e^{\beta_{N}\sigma_{1\;,\;1}\sigma_{1\;,\;N}}\prod_{k=0}^{n-1}e^{\beta_{k+1}\sigma_{1\;,\;N-k}\sigma_{1\;,\;N-(k+1)}}\right)\;, \end{array}$$

Further, (52) can be written in the form:

(53) $$\langle \sigma_{1,\;1}\sigma_{1,\;N-n}\rangle =\frac{1}{Z_{0}}{\left(2\sinh\frac{2J^{\prime }}{kT}\right)}^{\frac{LN}{2}}\left(\prod_{k=1}^{n}\underset{\beta_{k}\to 0}{\lim}\frac{\partial }{\partial \beta_{k}}\right)\underset{\beta_{N}\to 0}{\lim}\frac{\partial }{\partial \beta_{N}}\mathrm{Tr}\left(W\right),$$

where the matrix **W** belongs to the spin representative, and, by employing the method presented in Section 2, we can obtain the exact expressions of $\langle \sigma_{l,\;1}\sigma_{l,\;N}\rangle$, $\langle \sigma_{l,\;1}\sigma_{l,\;N-1}\rangle$, $\langle \sigma_{l,\;1}\sigma_{l,\;N-2}\rangle \;,\;\cdots$.

However, to save space, here we no longer discuss $\langle \sigma_{l,\;1}\sigma_{l,\;N-1}\rangle$, $\langle \sigma_{l,\;1}\sigma_{l,\;N-2}\rangle \;,\;\cdots$, but only consider $\langle \sigma_{l,\;1}\sigma_{l,\;N}\rangle$, for which a closed formula was given by Reference [12]:

(54) $$\langle \sigma_{1,\;1}\sigma_{1,\;N}\rangle =\frac{1}{Z_{0}}{\left(2\sinh\frac{2J^{\prime }}{kT}\right)}^{\frac{LN}{2}}\underset{\phi \to 0}{\lim}\frac{\partial Y}{\partial \phi },$$

(55) $$\begin{array}{ll} Y= & \frac{1}{2}\left(\prod_{l=1}^{\left[(N+1)/2\right]}2\cosh\frac{\chi_{2(l-1)}^{\left(+\right)}}{2}\right)\left(\prod_{m=1}^{\left[N/2\right]}2\cosh\frac{-\chi_{2m-1}^{\left(+\right)}}{2}\right)+\frac{1}{2}\left(\prod_{l=1}^{\left[(N+1)/2\right]}2\sinh\frac{\chi_{2(l-1)}^{\left(+\right)}}{2}\right)\left(\prod_{m=1}^{\left[N/2\right]}2\sinh\frac{-\chi_{2m-1}^{\left(+\right)}}{2}\right)\\ & +\frac{1}{2}\left(\prod_{l=1}^{\left[(N+1)/2\right]}2\cosh\frac{\chi_{2(l-1)}^{\left(-\right)}}{2}\right)\left(\prod_{m=1}^{\left[N/2\right]}2\cosh\frac{-\chi_{2m-1}^{\left(-\right)}}{2}\right)-\frac{1}{2}\left(\prod_{l=1}^{\left[(N+1)/2\right]}2\sinh\frac{\chi_{2(l-1)}^{\left(-\right)}}{2}\right)\left(\prod_{m=1}^{\left[N/2\right]}2\sinh\frac{-\chi_{2m-1}^{\left(-\right)}}{2}\right)\;, \end{array}$$

where $\chi_{2(l-1)}^{\left(\pm \right)}$ and $\chi_{2m-1}^{\left(\pm \right)}$ are determined by:

(56) $$e^{\chi_{2(l-1)}^{\left(\pm \right)}}=\tau_{2(l-1)}^{\left(\pm \right)}\left(l=1,\;2,\;\cdots ,\;\left[\frac{N+1}{2}\right]\right){;\;e}^{\chi_{2m-1}^{\left(\pm \right)}}=\tau_{2m-1}^{\left(\pm \right)}\left(m=1,\;2,\;\cdots ,\;\left[\frac{N}{2}\right]\right)\;,$$

$\tau_{n}^{\left(\pm \right)}$ (n=1, 2, ⋯, N) are *N* roots of the *N*-th order algebraic equation $F_{\pm }(\tau )=0$, where

(57) $$\begin{array}{ll} F_{\pm }(\tau )= & \left(\prod_{l=1}^{\left[(N+1)/2\right]}\left(\tau {\;e}^{-L\gamma_{2(l-1)}}-1\right)\right)\left(\prod_{m=1}^{\left[N/2\right]}\left(\tau {\;e}^{L\gamma_{2m-1}}-1\right)\right)\\ & \mp \tanh\phi \;\left(\prod_{l=1}^{\left[(N+1)/2\right]}\left(\tau {\;e}^{-L\gamma_{2(l-1)}}-1\right)\right)\left(\prod_{m=1}^{\left[N/2\right]}\left(\tau {\;e}^{L\gamma_{2m-1}}-1\right)\right)\\ & \mp 4\tanh\phi \sum_{n=1}^{\left[(N+1)/2\right]}\left(\Omega_{2(n-1)}^{2}\left(\prod_{\begin{array}{l} l=1\\ l\neq n \end{array}}^{\left[(N+1)/2\right]}\left(\tau {\;e}^{-L\gamma_{2(l-1)}}-1\right)\right)\left(\prod_{m=1}^{\left[N/2\right]}\left(\tau {\;e}^{L\gamma_{2m-1}}-1\right)\right)\right)\\ & \mp 4\tanh\phi \sum_{n=1}^{\left[N/2\right]}\left(\Omega_{2n-1}^{2}\left(\prod_{l=1}^{\left[(N+1)/2\right]}\left(\tau {\;e}^{-L\gamma_{2(l-1)}}-1\right)\right)\left(\prod_{\begin{array}{l} m=1\\ m\neq n \end{array}}^{\left[N/2\right]}\left(\tau {\;e}^{L\gamma_{2m-1}}-1\right)\right)\right)\;, \end{array}$$

where

(58) $$\Omega_{n-1}=\sinh2K^{\prime }\cosh K\sqrt{\frac{1-x_{n-1}^{2}}{N\sinh^{2}\gamma_{n-1}+\cosh\gamma_{r-1}\cosh2K^{\prime }-\cosh2K}}\;\left(n=1,\;2,\;\cdots ,\;N\right)$$

are the normalization constants of the eigenvectors of a rotation matrix [12], as N→∞. Thus, according to (47)~(51), we obtain:

(59) $$\begin{array}{l} \underset{N\to \infty }{\lim}\Omega_{n-1}\approx \frac{1}{\sqrt{N}}\sin\frac{(n-1)\pi }{N}\frac{\sinh2K^{\prime }\cosh K}{\sinh\gamma_{n-1}}\;(n=2,\;3,\;\cdots ,\;N)\;,\\ \underset{N\to \infty }{\lim}\Omega_{0}^{2}\approx \left\{ \begin{array}{l} \frac{1}{N}\sin^{2}\frac{\pi }{N}\frac{\sinh^{2}2K^{\prime }\cosh^{2}K}{\sinh^{2}2(K^{\prime }-K)}\;,\;T\geq T_{c}\;,\;K^{\prime }>K\;;\\ \frac{1}{N}\frac{\sinh^{2}2K^{\prime }\cosh^{2}K}{\sinh^{2}2K}\;,\;T\geq T_{c}\;,\;K^{\prime }=K\;;\\ \frac{\cosh2K-\cosh2K^{\prime }}{4\sinh^{2}K}\;.\;T<T_{c}\;. \end{array}\right. \end{array}$$

### 3.3. An Exact Expression of $\langle \sigma_{1,\;1}\sigma_{1,\;N}\rangle$ on a Finite Lattice

In Reference [12], all roots of the equation $F_{\pm }(\tau )=0$ are obtained by correcting to the $e^{-LC_{0}}$ order of magnitude ($C_{0}$ is a positive constant). These approximate roots can lead to the exact expression of $\langle \sigma_{l,\;1}\sigma_{l,\;N}\rangle$ in the thermodynamic limit, since $\underset{L\to \infty }{\lim}e^{-LC_{0}}=0$, but cannot lead to the exact expression of $\langle \sigma_{l,\;1}\sigma_{l,\;N}\rangle$ on a finite lattice. Hence, the expression of $\langle \sigma_{l,\;1}\sigma_{l,\;N}\rangle$ presented in Reference [12] is only an approximate result.

On the other hand, similar to the analysis in Section 2.5, the operator $\underset{\phi \to 0}{\lim}\frac{\partial }{\partial \phi }$ in (54) allows us to ignore all terms whose order is higher than $\phi^{1}(=\phi )$ in all roots of the equation $F_{\pm }(\tau )=0$. Hence, to obtain the exact expression of $\underset{\phi \to 0}{\lim}\frac{\partial Y}{\partial \phi }$, we need only to find all roots of the equation $F_{\pm }(\tau )=0$ corrected to the $\phi^{1}(=\phi )$ order, The corresponding calculations are in fact simpler than those required of find the roots corrected to the $e^{-LC_{0}}$ order of magnitude in Reference [12]; concretely, we obtain:

$$\chi_{2(l-1)}^{\left(\pm \right)}\approx L\gamma_{2(l-1)}\pm 4\Omega_{2(l-1)}^{2}\tanh\phi ,\;\chi_{2m-1}^{\left(\pm \right)}\approx -L\gamma_{2m-1}\pm 4\Omega_{2m-1}^{2}\tanh\phi .$$

Substituting the above results into (55), we obtain Y correcting to $\tanh\phi \left(\approx \phi^{1}\right)$ order:

$$\begin{array}{ll} Y\approx & \frac{1}{2}\left(\prod_{l=1}^{\left[(N+1)/2\right]}2\cosh\frac{L\gamma_{2(l-1)}+4\Omega_{2(l-1)}^{2}\tanh\phi }{2}\right)\left(\prod_{m=1}^{\left[N/2\right]}2\cosh\frac{L\gamma_{2m-1}-4\Omega_{2m-1}^{2}\tanh\phi }{2}\right)\\ & +\frac{1}{2}\left(\prod_{l=1}^{\left[(N+1)/2\right]}2\sinh\frac{L\gamma_{2(l-1)}+4\Omega_{2(l-1)}^{2}\tanh\phi }{2}\right)\left(\prod_{m=1}^{\left[N/2\right]}2\sinh\frac{L\gamma_{2m-1}-4\Omega_{2m-1}^{2}\tanh\phi }{2}\right)\\ & +\frac{1}{2}\left(\prod_{l=1}^{\left[(N+1)/2\right]}2\cosh\frac{L\gamma_{2(l-1)}-4\Omega_{2(l-1)}^{2}\tanh\phi }{2}\right)\left(\prod_{m=1}^{\left[N/2\right]}2\cosh\frac{L\gamma_{2m-1}+4\Omega_{2m-1}^{2}\tanh\phi }{2}\right)\\ & -\frac{1}{2}\left(\prod_{l=1}^{\left[(N+1)/2\right]}2\sinh\frac{L\gamma_{2(l-1)}-4\Omega_{2(l-1)}^{2}\tanh\phi }{2}\right)\left(\prod_{m=1}^{\left[N/2\right]}2\sinh\frac{L\gamma_{2m-1}+4\Omega_{2m-1}^{2}\tanh\phi }{2}\right). \end{array}$$

Substituting the above result and (41) into (54), we obtain the exact expression of $\langle \sigma_{1,\;1}\sigma_{1,\;N}\rangle$ of the model on a finite lattice:

(60) $$\langle \sigma_{1,\;1}\sigma_{1,\;N}\rangle =2\left(\sum_{l=1}^{\left[(N+1)/2\right]}\Omega_{2(l-1)}^{2}\coth\frac{L\gamma_{2(l-1)}}{2}-\sum_{m=1}^{\left[N/2\right]}\Omega_{2m-1}^{2}\coth\frac{L\gamma_{2m-1}}{2}\right)\left(\prod_{n=1}^{N}\tanh\frac{L\gamma_{n-1}}{2}\right)$$

Although the whole calculation process is complex, the final result (60) is simple.

### 3.4. The Expression of $\langle \sigma_{1,\;1}\sigma_{1,\;N}\rangle$ in the Thermodynamic Limit

To derive the expression of $\langle \sigma_{1,\;1}\sigma_{1,\;N}\rangle$ in the thermodynamic limit, we first discuss some properties of $\gamma_{n-1}$ (n=1, 2, ⋯, N).

For $\gamma_{n-1}$ (n=2, 3, ⋯, N) given by (47), we have:

(61) $$\underset{L\to \infty }{\lim}L\gamma_{n-1}=\infty ,\;\underset{L\to \infty }{\lim}\tanh\frac{L\gamma_{n-1}}{2}=\underset{L\to \infty }{\lim}\coth\frac{L\gamma_{n-1}}{2}=1\;\left(n=2,\;3,\;\cdots ,\;N\right).$$

As for $\gamma_{0}$, when $T\geq T_{c}$, from (51) we see that (61) still holds for $\gamma_{0}$; however, when $T<T_{c}$, from the last expression in (51) we see that maybe $\underset{L\to \infty }{\lim}L\gamma_{0}=\infty$ does not hold. For example, if $L=N^{a}$, where *a* is a positive integer, then:

$$\underset{L\to \infty }{\lim}L\gamma_{0}=\underset{N\to \infty }{\lim}N^{a}\cdot 2{\left(\frac{\tanh K^{\prime }}{\tanh K}\right)}^{N}\csc h2K\left(\cosh2K-\cosh2K^{\prime }\right)=0,$$

since now $0<\frac{\tanh K^{\prime }}{\tanh K}<1$, for 0<b<1, $\underset{N\to \infty }{\lim}N^{a}b^{N}=0$. Hence, for $\gamma_{0}$ we have:

(62) $$\underset{L\to \infty \;,\;N\to \infty }{\lim}\;L\gamma_{0}=\left\{ \begin{array}{l} \infty \;,\;T\geq T_{c}\;;\\ 0\;,\;T<T_{c}\;, \end{array}\right.\;\underset{L\to \infty \;,\;N\to \infty }{\lim}\;\tanh\frac{L\gamma_{0}}{2}=\left\{ \begin{array}{l} 1\;,\;T\geq T_{c}\;;\\ 0\;,\;T<T_{c}\;, \end{array}\right.\;\left(L=N^{a}\right)$$

According to the above discussions, to obtain the expression of $\langle \sigma_{1,\;1}\sigma_{1,\;N}\rangle$ in the thermodynamic limit, we first write (60) in the form:

$$\begin{array}{ll} \langle \sigma_{1\;,\;1}\sigma_{1\;,\;N}\rangle = & 2\Omega_{0}^{2}\left(\prod_{n=2}^{N}\tanh\frac{L\gamma_{n-1}}{2}\right)\\ & +2\tanh\frac{L\gamma_{0}}{2}\left(\sum_{l=2}^{\left[(N+1)/2\right]}\Omega_{2(l-1)}^{2}\coth\frac{L\gamma_{2(l-1)}}{2}-\sum_{m=1}^{\left[N/2\right]}\Omega_{2m-1}^{2}\coth\frac{L\gamma_{2m-1}}{2}\right)\left(\prod_{n=2}^{N}\tanh\frac{L\gamma_{n-1}}{2}\right)\;, \end{array}$$

as L→∞, according to (61), the above expression becomes:

(63) $$\underset{L\to \infty }{\lim}\langle \sigma_{1,\;1}\sigma_{1,\;N}\rangle \approx 2\Omega_{0}^{2}+2\tanh\frac{L\gamma_{0}}{2}\left(\sum_{l=2}^{\left[(N+1)/2\right]}\Omega_{2(l-1)}^{2}-\sum_{m=1}^{\left[N/2\right]}\Omega_{2m-1}^{2}\right)\;.$$

When $T\geq T_{c}$, according to (59) and (62), Equation (63) becomes:

$$\underset{L\to \infty }{\lim}\langle \sigma_{1,\;1}\sigma_{1,\;N}\rangle \approx 2\left(\sum_{l=1}^{\left[(N+1)/2\right]}\Omega_{2(l-1)}^{2}-\sum_{m=1}^{\left[N/2\right]}\Omega_{2m-1}^{2}\right)\;,$$

Further, as N→∞,

(64) $$\sum_{l=1}^{\left[(N+1)/2\right]}\Omega_{2(l-1)}^{2}\approx \sum_{m=1}^{\left[N/2\right]}\Omega_{2m-1}^{2}\approx \frac{1}{2}\sum_{n=1}^{N}\Omega_{n-1}^{2},$$

hence, for this case $\underset{L\to \infty \;,\;N\to \infty }{\lim}\langle \sigma_{1,\;1}\sigma_{1,\;N}\rangle =0$.

When $T<T_{c}$, according to (62) and (64), the second term in (63) vanishes, and (63) thus becomes $\underset{L\to \infty }{\lim}\langle \sigma_{1,\;1}\sigma_{1,\;N}\rangle \approx 2\Omega_{0}^{2}$, where $\Omega_{0}^{2}$ is given by the last expression in (59) as N→∞.

Summarizing the above results, in the thermodynamic limit, if $L=N^{a}$, then (60) becomes:

$$\underset{L\to \infty \;,\;N\to \infty }{\lim}\langle \sigma_{1,\;1}\sigma_{1,\;N}\rangle =\;\left\{ \begin{array}{ll} 0, & T\geq T_{c}\;;\\ \frac{\cosh2K-\cosh2K^{\prime }}{2\sinh^{2}K}, & T<T_{c}\;. \end{array}\right.$$

The above result was obtained in Reference [12] in terms of an approximate result of $\langle \sigma_{1,\;1}\sigma_{1,\;N}\rangle$. Some further discussions about the above result can be found in Reference [12].

From the above discussions, it is revealed how the changes of the values of $\gamma_{n-1}$ (n=1, 2, ⋯, N), especially the change of the value of $\gamma_{0}$, lead to the change of $\underset{L\to \infty \;,\;N\to \infty }{\lim}\langle \sigma_{1,\;1}\sigma_{1,\;N}\rangle$ when the system crosses its critical temperature $T_{c}$, as well as how the long-range order emerges as the temperature decreases.

## Figures and Tables

**Table 1:** The eigenvalues and eigenvectors of $H^{\left(0\right)}+\sinh2\phi_{1}H^{\left(1\right)}$ corrected to the $\phi_{1}^{1}(=\phi_{1})$ order.

Eigenvalue ${\left\{\;\tau^{\left(1\right)}\right\}}^{}$	Eigenvector ${\left\{\;\Psi^{\left(1\right)}\right\}}^{}$
$e^{\pm \left(L\gamma_{m}^{\left(↑↓\right)}+\alpha_{m,↑↓}^{\left(1\right)}\right)}$	$\frac{1}{\sqrt{2}}\left(\Psi_{m,\pm }^{\left(0\right)}-\Psi_{m^{\prime },\pm }^{\left(0\right)}\right)-\frac{\sinh2\phi_{1}}{2M}\Delta \Psi_{m,\pm ,↑↓,I}^{\left(0\right)}$
$e^{\pm \left(L\gamma_{m}^{\left(↑↓\right)}-\beta_{m,↑↓}^{\left(1\right)}\right)}$	$\frac{1}{\sqrt{2}}\left(\Psi_{m,\pm }^{\left(0\right)}+\Psi_{m^{\prime },\pm }^{\left(0\right)}\right)-\frac{\sinh2\phi_{1}}{2M}\Delta \Psi_{m,\pm ,↑↓,\mathrm{II}}^{\left(0\right)}$
$e^{\pm \left(L\gamma_{M}^{\left(↓\right)}+\delta_{M}^{\left(1\right)}\right)}$	$\Psi_{M,\pm }^{\left(0\right)}-\frac{\sinh2\phi_{1}}{4M}\Delta \Psi_{M,\pm ,↓}^{\left(0\right)}$
$e^{\pm \left(L\gamma_{2M}^{\left(↓\right)}+\delta_{2M}^{\left(1\right)}\right)}$	$\Psi_{2M,\pm }^{\left(0\right)}-\frac{\sinh2\phi_{1}}{4M}\Delta \Psi_{2M,\pm ,↓}^{\left(0\right)}$
